# *De novo* transcriptome sequencing and gene expression profiling of *Elymus nutans* under cold stress

**DOI:** 10.1186/s12864-016-3222-0

**Published:** 2016-11-04

**Authors:** Juanjuan Fu, Yanjun Miao, Linhui Shao, Tianming Hu, Peizhi Yang

**Affiliations:** 1 Department of grassland science, College of Animal Science and Technology, Northwest A&F University, Yangling, Shaanxi 712100 China; 2 College of Plant Science, Agriculture and Animal Husbandry College of Tibet University, Linzhi, Tibet 860000 China; 3 Institute of Animal Sciences, Chinese Academy of Agricultural Sciences, Beijing, 100101 China

**Keywords:** Cold stress, Co-expression, Dehydrin, Species-specificity, Transcriptome

## Abstract

**Background:**

*Elymus nutans* Griseb., is an important alpine perennial forage of Pooideae subfamily with strong inherited cold tolerance. To get a deeper insight into its molecular mechanisms of cold tolerance, we compared the transcriptome profiling by RNA-Seq in two genotypes of *Elymus nutans* Griseb. the tolerant Damxung (DX) and the sensitive Gannan (GN) under cold stress.

**Results:**

The new *E. nutans* transcriptomes were assembled and comprised 200,520 and 181,331 transcripts in DX and GN, respectively. Among them, 5436 and 4323 genes were differentially expressed in DX and GN, with 170 genes commonly expressed over time. Early cold responses involved numerous genes encoding transcription factors and signal transduction in both genotypes. The AP2/EREBP famliy of transcription factors was predominantly expressed in both genotypes. The most significant transcriptomic changes in the later phases of cold stress are associated with oxidative stress, primary and secondary metabolism, and photosynthesis. Higher fold expressions of fructan, trehalose, and alpha-linolenic acid metabolism-related genes were detected in DX. The DX-specific dehydrins may be promising candidates to improve cold tolerance. Twenty-six hub genes played a central role in both genotypes under cold stress. qRT-PCR analysis of 26 genes confirmed the RNA-Seq results.

**Conclusions:**

The stronger transcriptional differentiation during cold stress in DX explains its better cold tolerance compared to GN. The identified fructan biosynthesis, alpha-linolenic acid metabolism, and DX-specific dehydrin-related genes may provide genetic resources for the improvement of cold-tolerant characters in DX. Our findings provide important clues for further studies of the molecular mechanisms underlying cold stress responses in plants.

**Electronic supplementary material:**

The online version of this article (doi:10.1186/s12864-016-3222-0) contains supplementary material, which is available to authorized users.

## Background

*Elymus nutans* Griseb., is an important alpine perennial forage of Pooideae subfamily, and mainly distributed on the Qinghai-Tibet Plateau and in the Himalayas [1]. Due to its high adaptability, good source of nutrients, high yield, and resistance to various abiotic stresses, such as low temperature, high radiation, and large diurnal temperature changes, it is often used for ecological restoration and the construction of artificial grasslands [1, 2]. To understand the mechanism of how *E. nutans* resists low temperature limitations, a better understanding of the gene expression profile of *E. nutans* under cold stress is imperative. This is because it could be an ideal model to study cold tolerance mechanisms for the purpose of improving the quality of cold resistance in other cold sensitive plants.

Cold stress is a major abiotic factor that limits plant growth, development, survival and productivity [3]. To overcome this barrier, plants have evolved various adaptive mechanisms that trigger a cascade of events leading to changes in gene expression and subsequently to physiochemical modifications that enhance their cold tolerance [4]. The physiological response includes: induction of transient increases in Ca^2+^ and ABA levels [5, 6], alterations in lipid composition, increases in antioxidant levels, and the accumulation of osmoprotectants [7].

Gene-expression changes that have been induced by stress form a key components of the molecular mechanisms by which plants adapt to environmental challenges [8]. Numerous studies provide evidence of global changes in gene expression in response to cold stress [9–14] and these changes highlight the importance of transcriptional regulation in plant adaptation to cold stress. For example, cold stress induced around 2979 genes in sheepgrass, while more than 2 % of wheat genome showed different expression patterns in response to cold [10, 11]. Numerous cold regulated genes encode transcription factors or proteins are involved in transcription [15]. Among these, the C-repeat binding factor (CBF)/dehydration responsive element provides one of the most important pathways for cold response. Cold-induced CBFs, which are members of the APETALA2/ETHYLENE RESPONSE FACTOR (AP2/ERF) gene family, can activate cold response (COR) genes by binding to the cis-acting elements in their promoters [16]. Many of COR genes, such as dehydrin, encode cryoprotective proteins that protect plant cells against cold-induced damage [4]. In barley, several dehyrin related genes, *Dhn5*, *Dhn8* and *Dhn13* accumulate in response to cold [17]. However, so far, the cold stress-response mechanism in *E. nutans* remains to be identified.

To elucidate physiological and transcriptomic adaptive mechanisms in *E. nutans*, the wild DX and variety GN were exposed to 4 °C for different time courses. Our physiochemical study demonstrated that DX had better cold tolerance than GN. As seen by lower electrolyte leakage level, low lipid peroxidation, and the maintenance of higher photosynthetic activity under cold stress. Here, comparative transcriptome analyses were performed to provide insights into the molecular mechanisms of cold tolerance in *E. nutans*. Identification of cold-responsive genes encoding transcription factors, signal transduction, enzymes involved in primary and secondary metabolism as well as specific dehydrin genes provided a better understanding of the molecular mechanism underling the response to cold stress in this species.

## Results

### The DX genotype has a better cold tolerance compared with GN genotype

To elucidate the physiological responses of both genotypes exposed to cold stress, biomass and several important physiological indices were measured. Cold stress relatively reduced the plant fresh weight of GN but not of DX (Additional file 1: Figure S1a). Concurrent with growth inhibition, there was a lower relative water content in GN than DX exposed to 24 h and 5 d of cold treatment (Additional file 1: Figure S1b). Malondialdehyde (MDA), a product of lipid peroxidation, considered as an indicator of oxidative injury under abiotic and biotic stress [18]. MDA content and electrolyte leakage levels increased (*P* < 0.05) in both plants under 24 h and 5 d of cold stress, while a smaller increase in these values was observed in DX (Additional file 1: Figure S1c, d), indicating lower oxidative damage in DX under cold stress. DX accumulated (*P* < 0.05) higher proline than GN in both control and stressed conditions (Additional file 1: Figure S1e). These results suggest that the DX genotype was more cold-tolerant than GN genotype.

### Transcriptome assembly and annotation

To further elucidate the molecular mechanisms underlying differential cold tolerance in DX and GN, twenty-four cDNA libraries were created from three independent biological samples of four treatments (0, 3, 24 h, and 5d) and sequenced using the Illumina HiSeq™ 4000 sequencing platform. More than 65 M high-quality pair-end reads were retrieved after trimming for each RNA-seq sample. Sequencing was done on the Illumina platform generating paired end reads of 100 bp each. Cleaned reads were *de novo* assembled using Trinity software: a total of 200,520 and 181,331 transcripts with a N50 of 1809 and 1777 bp were generated in DX and GN genotypes, respectively (Additional file 2: Table S1). This indicates a high quality assembly. The size distribution of transcripts was shown in Additional file 3: Figure S2.

To validate and annotate the of assembled unigenes, using E-value < 1e-5, they were blast searched against seven public databases, including NR, NT, COG, GO, KEGG, Swissprot, and Interpro protein database. In total, 164,827 (82.2 %) and 150,545 (83.02 %) unigenes in the DX and GN assemblies were found in at least one of these databases (Additional file 4: Table S2). Overall, the unigene sequences were most similar to gene sequences from *Hordeum vulgare subsp. vulgare*, *Aegilops tauschii*, *Triticum urartu*, *Brachypodium distachyon* via BLASTx matches (Additional file 5: Figure S3).

To characterize the functional classifications of annotated unigenes, GO and KEGG analyses were performed to access the distributions of functional categories. A total of 62,654 (31.29 %) and 55,886 (30.82 %) unigenes were annotated in GO for DX and GN, respectively, and classified into 57 functional groups, including 23 groups in biological process, 17 in molecular function, and 17 in cellular components (Additional file 6: Figure S4). In total, 82,192 (40.99 %) unigeness in DX and 76,364 (42.11 %) in GN were assigned to 20 KEGG pathways. These unigenes were mainly involved in ‘Translation’, ‘Lipid metabolism’, and ‘Transport and catabolism’ (Additional file 7: Figure S5).

### Gene expression under cold stress

**Fig. 1:**
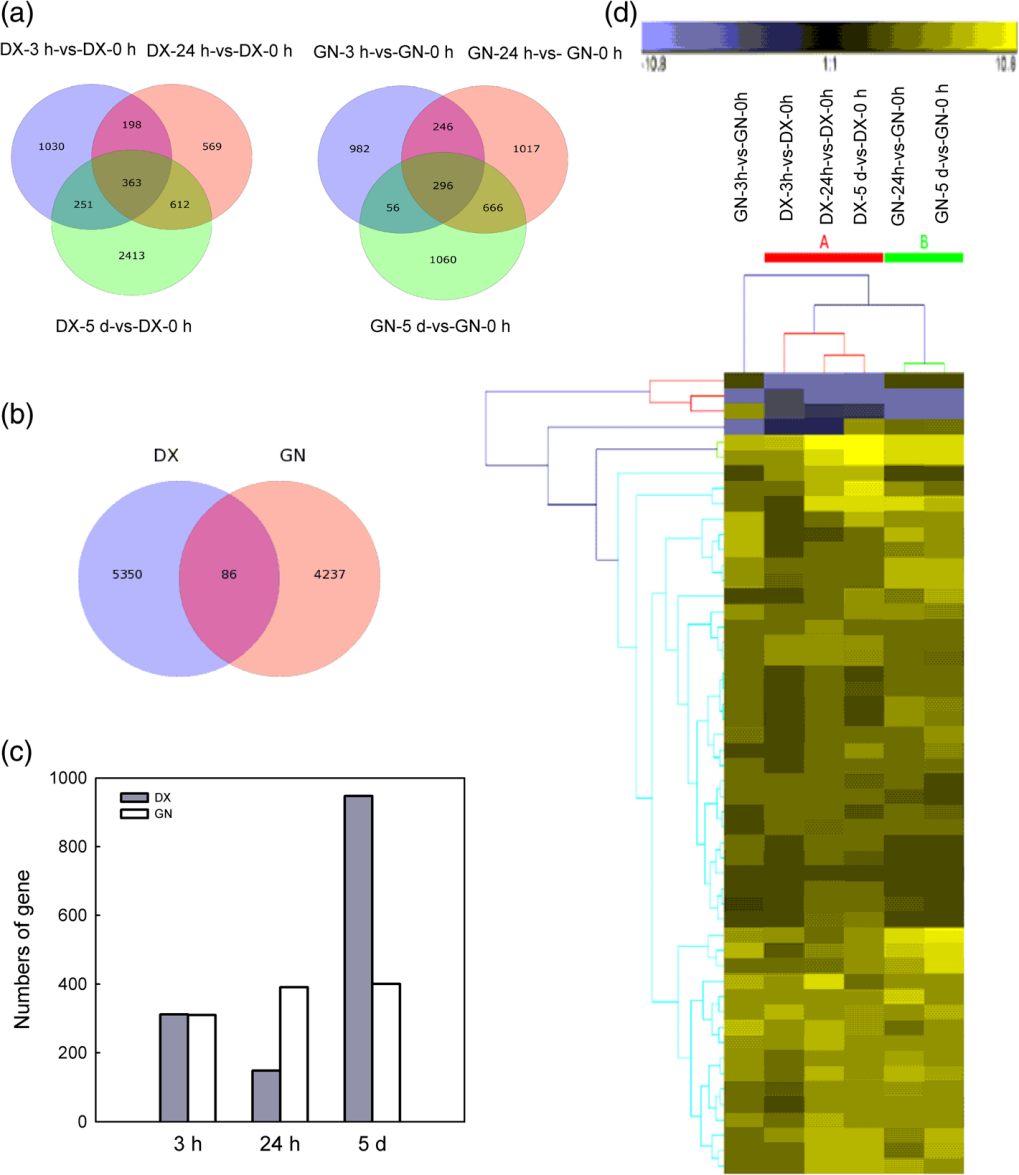
Transcriptional profiles of DX and GN genotypes after 0 to 5 d of cold exposure. **a** Venn diagram showing the number of differentially expressed genes (DEGs) in DX and GN genotypes under cold stress compared with control plants. **b** The number of DEGs common and specific for DX and GN during cold stress. **c** The number of DX and GN uniquely expressed genes among 4076 DEGs between the two genotypes. **d** Hierarchical clustering (HCL) analysis of the common expressed DEGs from the RNA-Seq in DX and GN (*yellow*, induced genes; *blue*, repressed genes)

Differential gene expression was analyzed relative to a control (0 h) grown under control conditions. To characterize the differentially expressed genes (DEGs) over the 5 d of cold exposure, a total of 5436 and 4323 DEGs (*P*-value ≥ 0.8 and |log2 (fold change)| >2) were identified and analyzed for the DX and GN genotypes, respectively (Fig. 1a; Additional file 8: Table S3). Most of the cold regulated DEGs are late-response genes. In DX, 2413 (44.4 %) unigenes were induced/repressed exclusively at 5 d of cold treatment, while only 1030 (18.9 %) and 569 (10.9 %) unigenes were specific at 3 or 24 h of cold treatment. A similar change in DEGs over time was observed in GN, starting from 1580 DEGs after 3 h to 2078 DEGs after 5 d of cold stress (Fig. 1a). For all comparisons in pairs, we identified 5350 DEGs in DX and 4237 in GN, and only 86 DEGs were commonly regulated for both genotypes (Fig. 1b), indicating that the cold-induced transcriptomic responses were largely genotype specific. A total of 4074 DEGs (*P*-value ≥ 0.8 and |log2 (fold change)| >2) were identified between the treated and control DX and GN plants (Additional file 9: Table S4). A total of 94 and 76 DEGs in the DX and GN genotypes represented 52 common response genes (8 persistently upregulated) at all time points. While 2451 genes were specifically regulated for each time point in DX and GN, illustrating a high species-specific transcriptomic plasticity in response to cold stress (Fig. 1c; Additional file 10: Table S5; Additional file 11: Table S6; Additional file 12: Table S7; Additional file 13: Table S8).

A hierarchical cluster analysis (HCL) was performed to characterize the expression patterns of the common expressed DEGs after cold exposure. A large proportion of DEGs in DX and GN followed a similar expression pattern after 24 h and 5 d of cold treatment, which clustered together. In contrast, expression profile of 3 h-exposed plants clustered separately, this indicated obvious differences in the global gene expression patterns at early and later phases of cold stress in the two genotypes (Fig. 1d).

### Pathways enrichment analysis of DEGs

Using the KEGG database, pathways displaying significant changes (Q value ≤ 0.05) in response to the cold treatment were identified in both genotypes (Additional file 14: Table S9). In the 3 h cold treatment sample, 21 and 16 KEGG pathways were significantly enriched in DX and GN, respectively. Among the top 5 enriched pathways, ‘mRNA surveillance pathway (ko03015)’, ‘circadian rhythm – plant (ko04712)’, and ‘RNA transport (ko03013)’ were common regulated in response to 3 h cold stress in both genotypes. The intermediate and late phases of the cold response were characterized by protective response through modulating cellular metabolic homeostasis. In the 24 h cold treatment sample the enriched pathway were similar to those at 5 d in both genotypes. In DX, ‘phenylpropanoid biosynthesis (ko00940)’, ‘cyanoamino acid metabolism (ko00460)’, and ‘galactose metabolism (ko00052)’ were the top three enriched pathways after 24 h and 5 d of cold stress. In GN, ‘glutathione metabolism (ko00480)’, ‘flavonoid biosynthesis (ko00941)’, ‘biosynthesis of secondary metabolites (ko01110)’, ‘phenylpropanoid biosynthesis (ko00940)’, and ‘flavone and flavonol biosynthesis (ko00944)’ were the most significantly enriched.

### Early gene-expression changes in response to cold stress

To gain insight into the functional categories of common and genotype-specific DEGs induced by 3 h of cold stress, GO term annotations were analyzed. For genes that were detected in the two genotypes at 3 h, the top enriched GO categories were invovled in ‘transcription factors (TFs)’ and ‘signal transduction’. Numerous genotype-specific TFs and signaling molecules were detected during early cold stress, indicating that DX and GN activated the downstream defense response through different signal transduction and transcriptional regulation pathways.

**Fig. 2:**
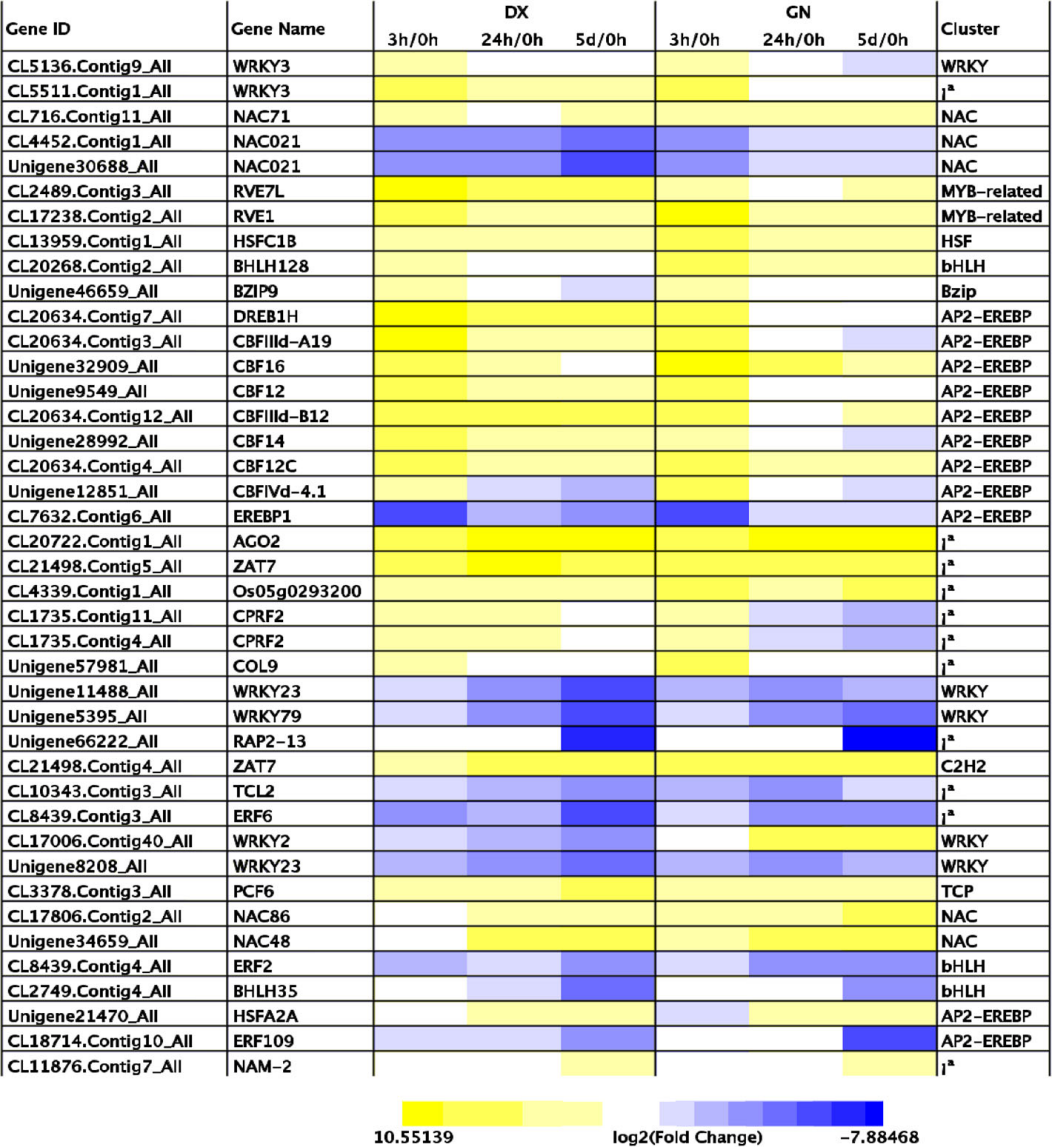
Heat map showing the expression profiles of transcription factor families in DX and GN genotypes. *Yellow* indicates the up-regulated genes and *blue* down-regulated genes

Transcription factor plays a key role in the regulation of upstream cold signal transduction which are capable of activating a cascade of downstream gene transcript [3]. A total of 118 genes encoding TFs belonging to 11 families were differentially expressed in both genotypes after 3 h of cold exposure (Additional file 15: Table S11). These TFs were asssociated with response to abiotic and biotic stresses and the regulation of developmental processes, including AP2-EREBP, basic helix–loop–helix (bHLH), MYB-related, MADS, NAC, basic leucine zipper (bZIP), cysteine-2/histidine-2 zinc finger proteins (C2C2)-Dof, and heat shock transcription factors (HSF). *CBFs* were the most represented subfamily with 40 members, including five DX-specific and 19 GN-specific genes. Additionally, other TFs such as bZIP, bHLH, NAC were cold-regulated in the two plants (Fig. 2). MADS, Sigma70-like, and Alfin-like subfamliy TFs were uniquely expressed in DX (Additional file 15: Table S11).

When suffering from unfavorable conditions, plants could trigger multiple stress-responsive signal transduction pathways that activate gene transcription and the downstream physiological adaptation [19]. Two DX-specific Ca^2+^ signaling-related genes, glutamate receptor 2.7 (*GLR2.7*) and calmodulin-binding transcription activator 4 (*CMTA4*), were induced during 3 h of cold stress and two GN-specific, glutamate receptor 3.4 (*GLR3.4*) and CBL-interacting protein kinase 7 (*CIPK7*) were induced during this time frame. Additionally, the up-regulation of calmodulin-like protein 3 (*CML3*) was higher in DX, suggesting *CML3* might contribute to higher cold tolerance in DX (Additional file 16: Table S12). Protein phosphorylation and dephosphorylation have been implicated in cold signal transduction, including 75 genes annotated as encoding protein kinases and phosphatases in both genotypes (Additional file 17: Table S12). Ten genes related to phytosulfokine receptor 2 were over-accumulated in both genotypes, indicating that phytosulfokine receptor signaling may play an important role in regulating the early cold signalings in *E. nutans*.

We also observed metabolic changes after 3 h of cold stress, indicated by the GO term ‘biosynthetic process’ (GO:0009058). For example, early changes involved in secondary metabolism processes, such as the induction of genes encoding flavonoid and lignin biosynthesis (CL3209.Contig3_All, CL8645.Contig8_All, Unigene11053_All, Unigene39201_All, etc) were detected after 3 h (Additional file 10: Table S5; Additional file 11: Table S6; Additional file 12; Table S7). Furthermore, lipid metabolism changed during cold stress, indicated by up-regulation of genes coding wax, suberin (CL14480.Contig22_All, CL1381.Contig2_All, CL2649.Contig4_All, etc), and fatty acid biosynthesis (Unigene82701_All, CL11835.Contig5_All). Genes coding sucrose: sucrose 1-fructosyltransferase (*1-SST*) indicated the need for the regulation of fructan biosynthesis. Moreover, the early response encompassed the accumulation of numberous cold responsive protein-coding genes (CL10923.Contig3_All, CL6160.Contig2_All, and Unigene38511_All, etc.).

In order to further identify specific metabolic pathways differentially affected by early cold stress in DX and GN leaves, a KEGG enrichment analysis (Q value ≤ 0.05) was performed. Several different metabolic pathways were affected by early cold stress in both genotypes, including mRNA surveillance pathway (ko03015)’, ‘circadian rhythm – plant (ko04712)’, ‘RNA transport (ko03013)’, and ‘biosynthesis of secondary metabolites (ko01110)’ (Additional file 18: Table S10). In the class of biosynthesis of secondary metabolites, most enzymes of stilbenoid, diarylheptanoid and gingerol biosynthesis (ko00945) and flavonoid biosynthesis (ko00941) were specifically induced in both genotypes. In addtion, seven genes of phenylpropanoid biosynthesis (ko00940) were specifically induced in DX in response to early cold stress. Specifically, most genes of starch and sucrose metabolism (ko00500), galactose metabolism (ko00052), and cutin, suberine and wax biosynthesis (ko00073) were induced in DX during the early cold stress (Additional file 18: Table S10).

### DEGs in response to intermediate and late phases of the cold stress

The intermediate and late phases of the cold response were characterized by increased lipid metabolism, oxidative stress, carbohydrate metabolism, secondary metabolism, and photosynthetic process.

**Fig. 3:**
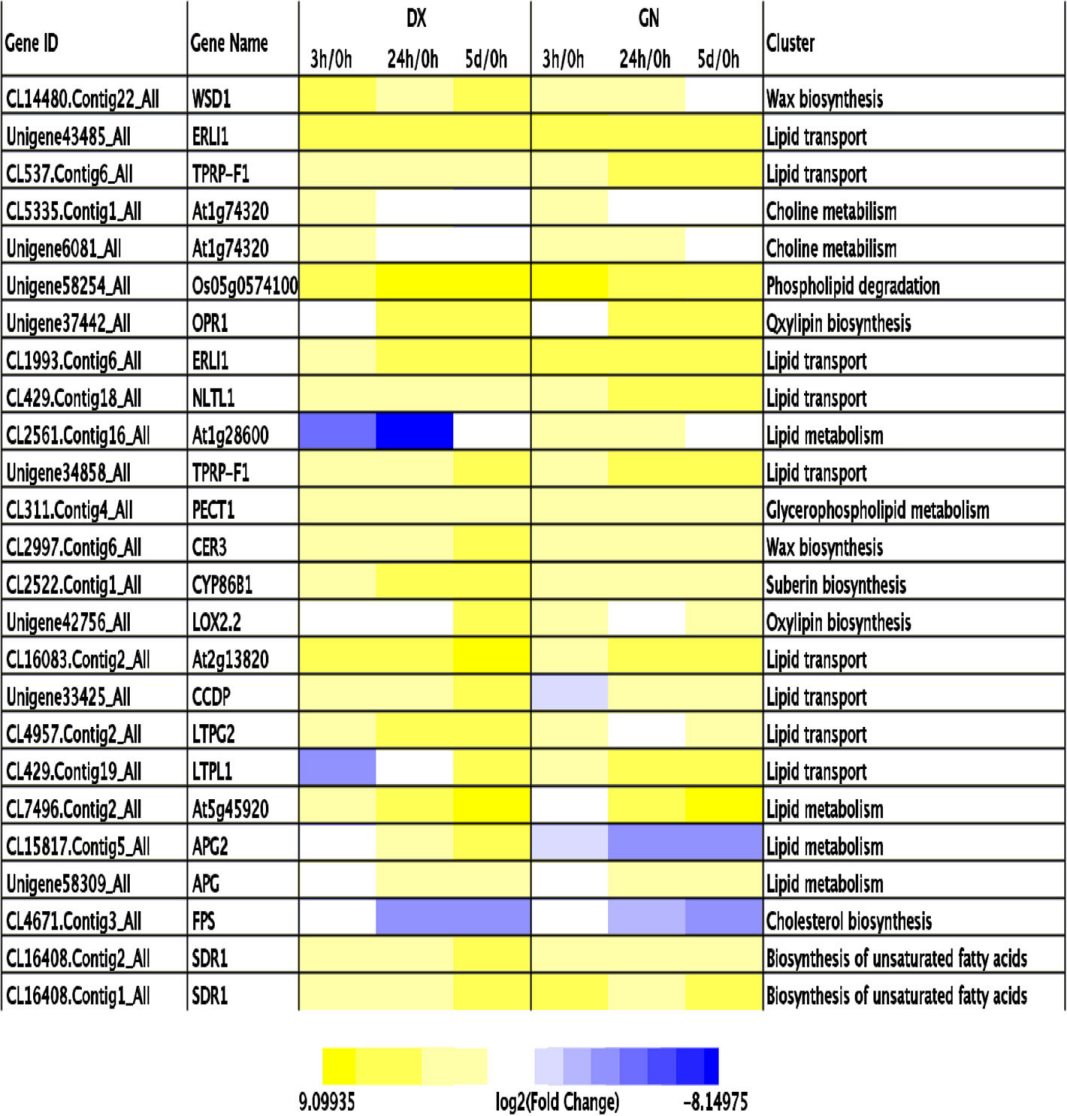
Heat map showing the expression profiles of lipid metabosim-related genes in DX and GN genotypes. *Yellow* indicates the up-regulated genes and *blue* down-regulated genes

‘Lipid metabolism process’ (GO:0006629) and ‘lipid transport’ (GO:0006869) were significantly induced in both genotypes during the intermediate and late stress treatment. A total of 99 lipid metabolism-related genes were identified, most of them involved in oxylipin, wax, cutin, suberin, sterol, and fatty acid biosynthetic and metabolic processes (Fig. 3; Additional file 16: Table S13). Genotype-specific regulation was prominent, as different genes—albeit with similar functions—were affected in the two genotypes. Genes encoding wax biosynthesis were strongly up-regulated at 24 h and 5 d in DX. Three oxylipin biosynthesis-related genes exhibited higher expression in DX, indicating oxylipin might contribute to better cold tolerance in DX. Notably, an approximately 10 fold increase in the gene encoding fatty acid desaturase (CL3006.Contig17_All) was observed after cold treatment at 5 d in DX (Additional file 16: Table S13).

**Fig. 4:**
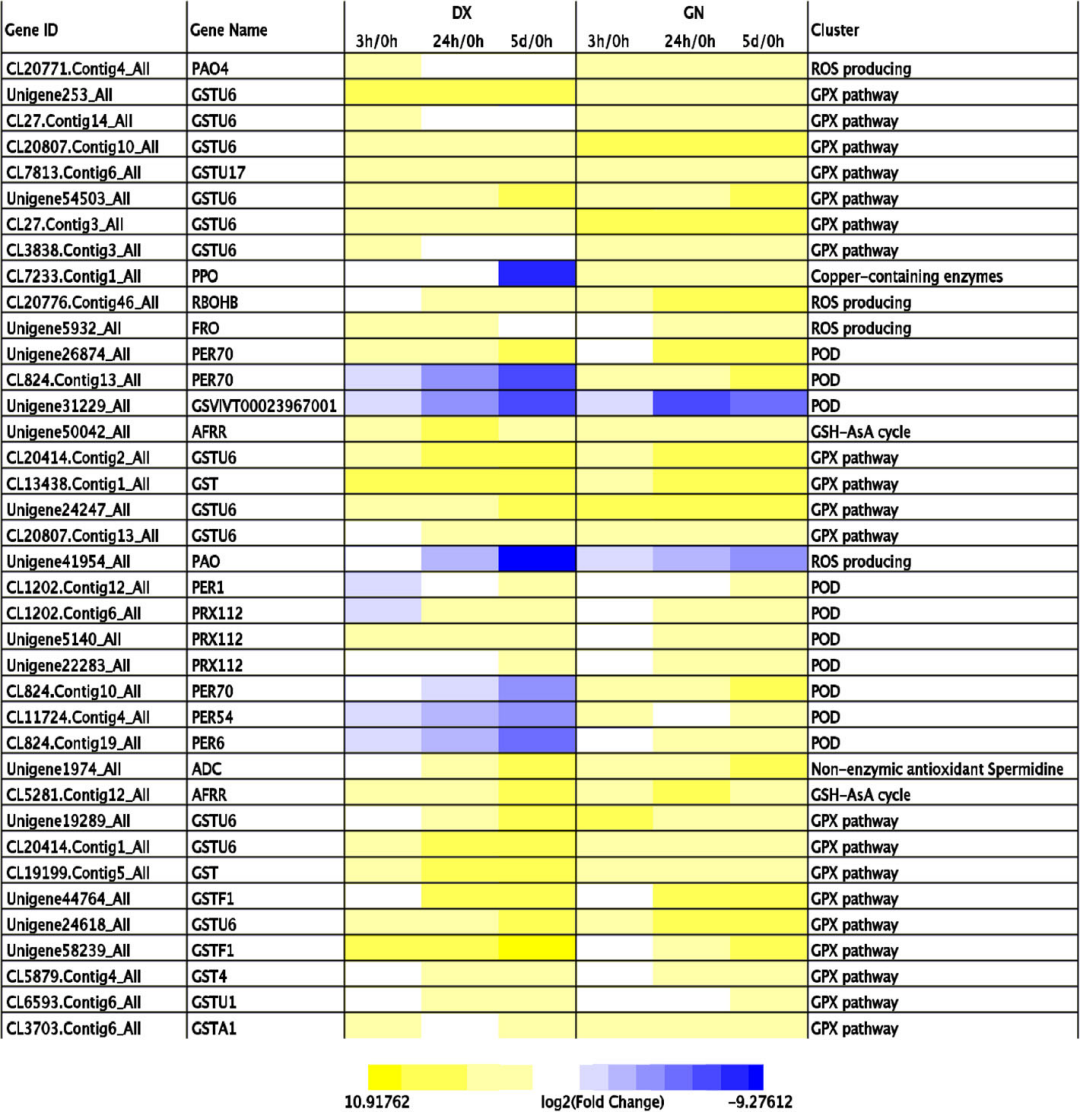
Heat map showing the expression profiles of ROS-related genes in DX and GN genotypes. *Yellow* indicates the up-regulated genes and *blue* down-regulated genes

Cold stress resulted in a higher reactive oxygen species (ROS) accumulation and lower antioxidant enzyme activities in GN compared with DX (Additional file 19: Figure S6), which might contribute to lower oxidative damage in DX. To further characterize the differences in ROS-related gene expression between tolerant and sensitive genotypes, cold-responsive genes related to ROS were analyzed. In all, 125 genes coding ROS producing and detoxification, including 29 in DX, 31 in GN and 65 in both genotypes (Additional file 20: Table S14), were identified as having significantly different expression profiles after late cold treatment. Consistent with higher production of ROS in GN, several genes coding respiratory burst oxidase protein and polyamine oxidase (CL20776.Contig4_All, CL20776.Contig46_All, Unigene5932_All), invovled in the generation of ROS, were more strongly induced by the late cold stress in GN than in DX (Fig. 4). Eighty-three DEGs were identified as encoding enzymes associated with ROS scavenging, including peroxidase (POD), catalase (CAT), ascorbate peroxidase (APX), monodehydroasorbate reductase (MDAR), glutathione S-transferase (GST), glutathione peroxidase (GPX), polyphenol oxidase (PPO), glutaredoxin, thioredoxin. Among the antioxidant enzymes we identified, GST and POD play the most important roles in scavenging ROS in both plants. Moreover, the induction of several genes involved in polyamine and tocopherol biosynthesis in both genotypes, were also noteworthy (Fig. 4; Additional file 20: Table S14).

**Fig. 5:**
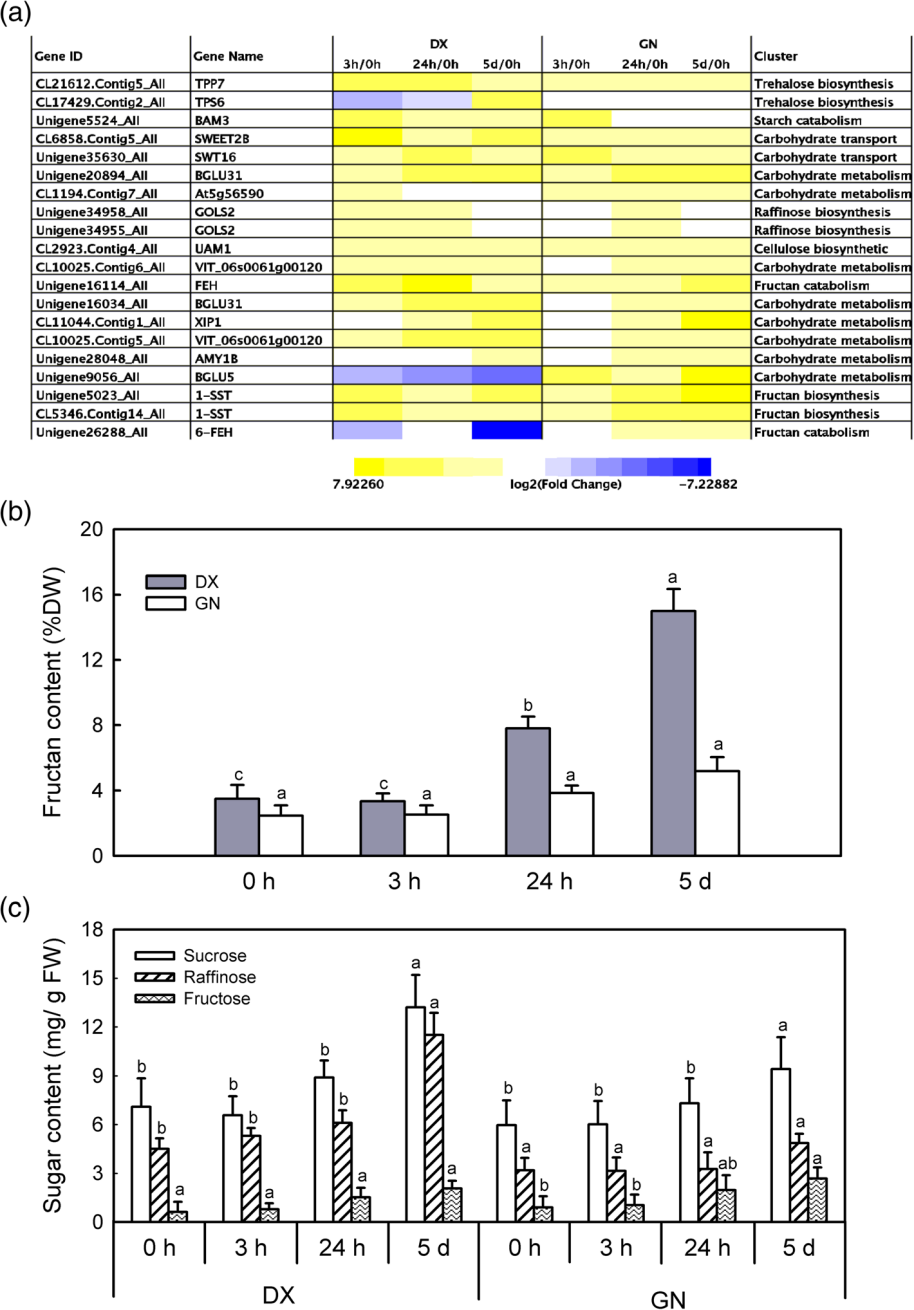
Carbohydrate metabosim in response to cold stress. **a** Heat map showing the expression profiles of genes related to carbohydrate metabosim in DX and GN genotypes. Yellow indicates the up-regulated genes and blue down-regulated genes. Changes in fructan (**b**), sucrose, fructose, and raffinose (**c**) content over time. Each value represents the mean of three replicates ± SE shown by the *vertical error bar*. The letters above the *bars* indicate a significant difference at the 0.05 level according to Duncan’s multiple range test

Carbohydrate metabolism was affected by cold stress in both genotypes (Additional file 21: Table S15). Fructan plays important roles in response to cold stress, corroborated by the induction of *1-SST* and differential expression of fructan 6-exohydrolase (*6-FEH*) in both plants (Fig. 5a, b). The fructan content was increased around two-fold in GN and five-fold in DX during 5 d of cold treatment. Consistent with increased concentrations in sucrose, fructose, and raffinose, enzymes such as sucrose synthase 1, beta-amylase, alpha-amylase isozyme C, and galactinol synthase 2 (Fig. 5; Additional file 21: Table S15), were induced in both plants. Additionally, cold stress increased the abundance of alpha, alpha-trehalose-phosphate synthase (*TPS6*) transcript by 5.2-fold in DX at 5 d under cold stress.

**Fig. 6:**
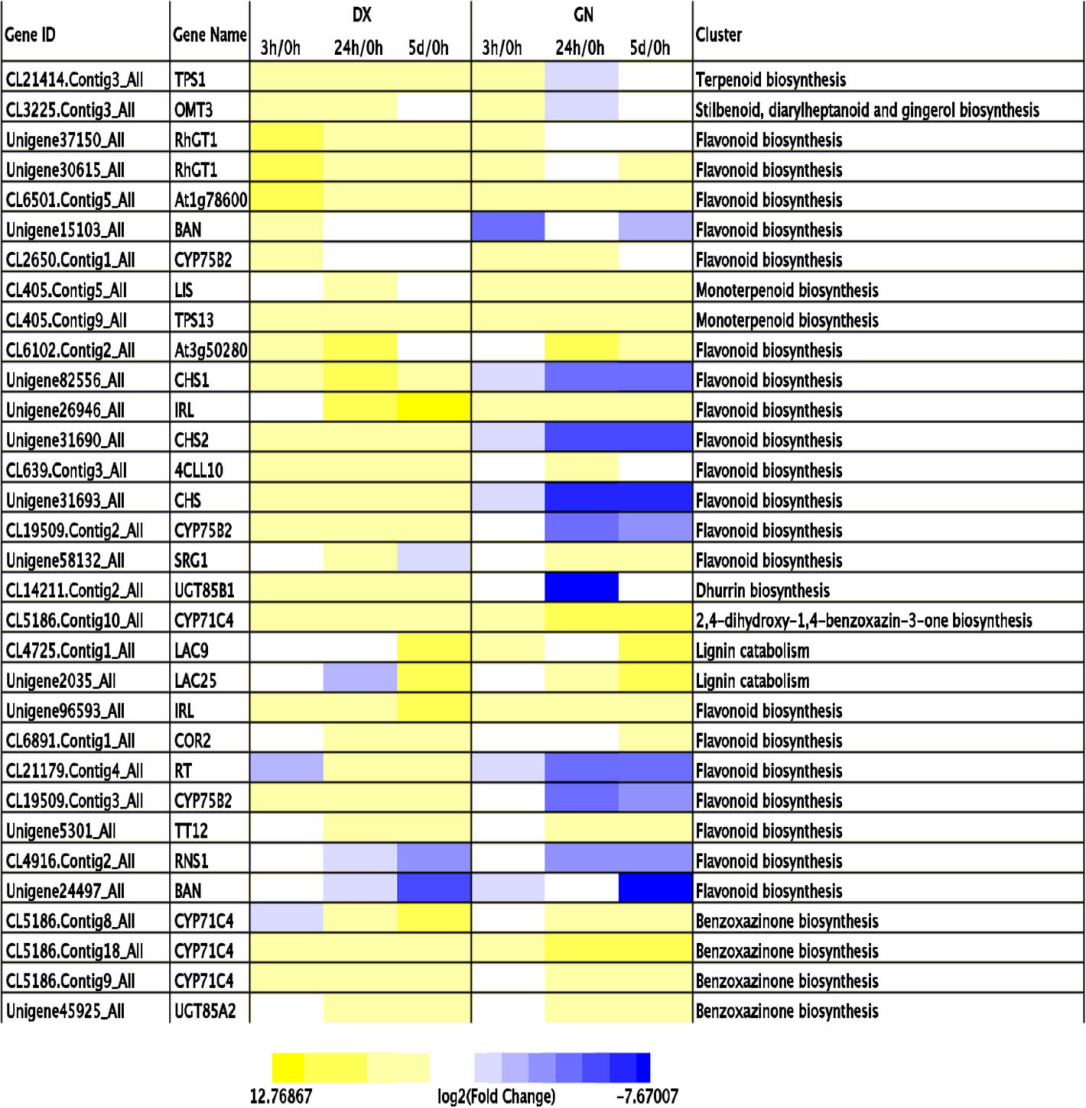
Heat map showing the expression profiles of secondary metabosim-related genes in DX and GN genotypes. Yellow indicates the up-regulated genes and blue down-regulated genes

The ‘secondary metabolism’ category also showed genotype-specificity in both plants. In all, 110 genes encoding secondary metabolism biosynthesis, including 44 DX specificity, 34 GN specificity and 32 commonly expressed (Additional file 22: Table S16). Flavonoids and stilbenes have ROS-scavenging activity that protects against oxidative damage and controls ROS levels under abiotic stresses [20]. Most of the flavonoid biosynthesis pathway responded late in the cold, with higher expression in DX than in GN. In the stilbenes biosynthesis pathway, one putative O-methyltransferase 2 (*OMT2*; Unigene44864_All) was upregulated by 9.6-fold in DX and trans-resveratrol di-O-methyltransferase (*ROMT*; CL13661.Contig2_All) was induced by 7.9-fold in GN. In DX tropane alkaloid, caffeine, menthol and paclitaxel biosynthesis were induced uniquely, this contrasted with GN in which benzylisoquinoline alkaloid biosynthesis was exclusively depressed. Lignin metabolism was significatly enriched in both genotypes. Altered pathways included, for example, the up-regulation of lignin catabolism and down-regulation of ligin biosynthesis in GN (Fig. 6).

**Fig. 7:**
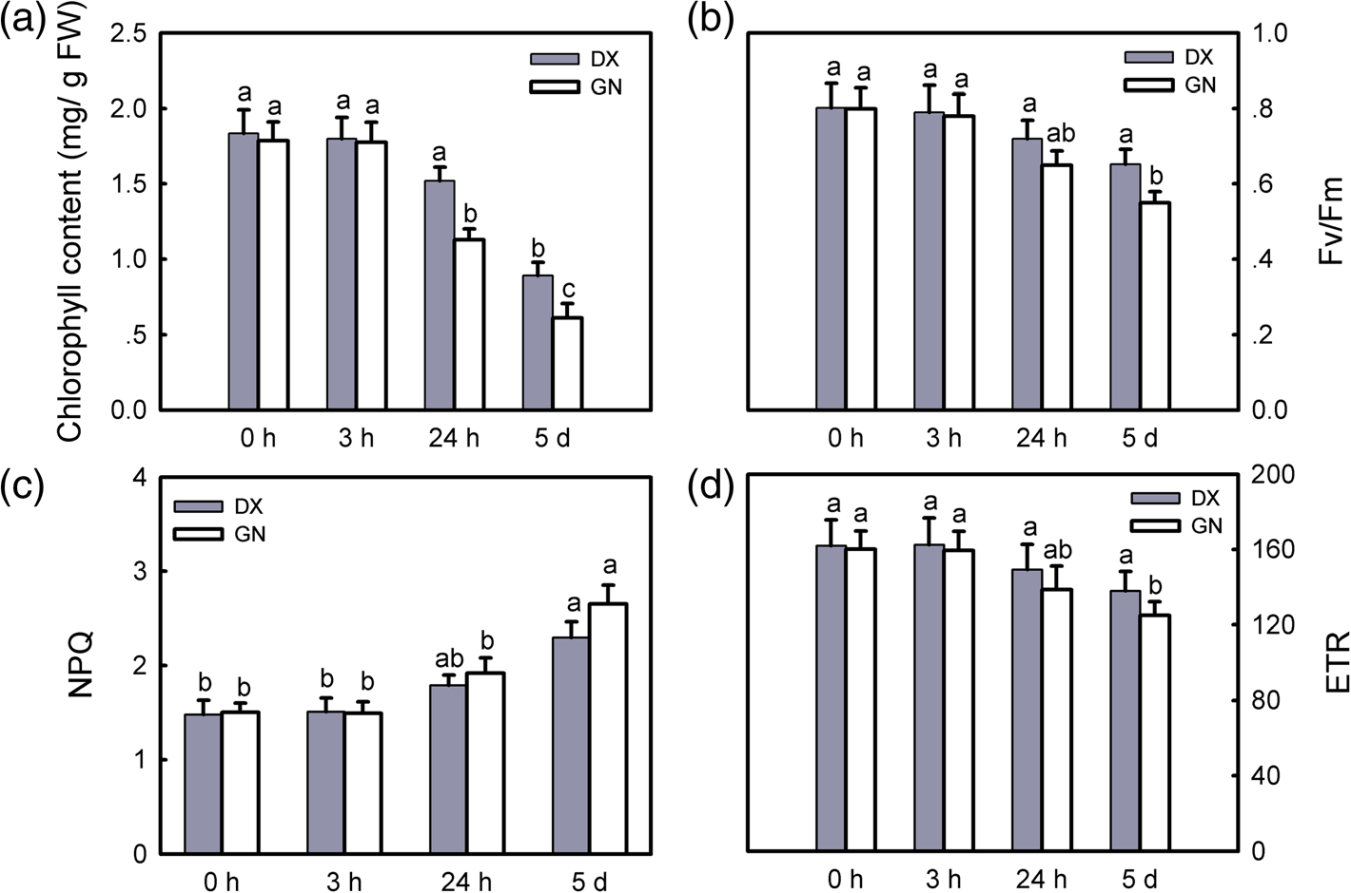
Changes in photosynthesis-related parameters in DX and GN under cold stress. **a** chlorophyll content, (**b**) the maximum photosystem II quantum yield (Fv/Fm), (**c**) apparent electron transport rate (ETR), and (**d**) non-photochemical quenching (NPQ). Each value represents the mean of three replicates ± SE shown by the *vertical error bar*. The letters above the *bars* indicate a significant difference at the 0.05 level according to Duncan’s multiple range test

Numerous genes encoding components of photosystem I (PSI) and PSII declined significantly during the intermediate and late phases of cold treatment. Over-represented GO terms describing sets of declining genes after 24 h and 5 d encompassed: ‘photosynthesis’ (GO:0015979), chloroplast thylakoid membrane (GO:0009535), and photosynthesis, light harvesting (GO:0009765) (Additional file 23: Table S17). Additionally, six genes encoding chlorophyll *a* biosynthesis significantly decreased in both genotypes, including protochlorophyllide reductase (*POR*) and glutaminyl-tRNA synthetase (*HEMA*). Indeed, chlorophyll content had a marked reduction in both plants under cold stress, showing a greater reduction in GN compared to DX (Fig. 7a). Transcriptome analysis showed that PSII was inhibited more severely in GN (Additional file 23: Table S17). To confirm this, PAM was used to determine the maximum quantum efficiency of PSII (Fv/Fm), apparent electron transport rate (ETR), and non-photochemical quenching (NPQ). During cold stress, Fv/Fm and ETR decreased, whereas NPQ increased in both plants. DX maintained higher Fv/Fm, ETR, and lower NPQ than GN under cold stress (Fig. 7).

Genes involved in specific metabolic pathways and cellular response processes in both genotypes were further analysed using KEGG enrichment analysis. The intermediate and late phases of the cold response were characterized by protective response through modulating cellular metabolic homeostasis, which is reflected by the largest numbers of metabolic pathways (ko01100) in both genotypes in response to the prolonged cold stress. A variety of different biochemical pathways were affected by cold stress in both plants, including cartbohydratre metabolism, energy generation, lipid metabolism, and secondary metabolism (Additional file 18: Table S10). In the category of lipid metabolism, genes of sterol biosynthetic process (ko00100), alpha-linolenic acid metabolism (ko00592), and most cutin, suberine and wax biosynthesis (ko00073) were induced in DX during the late cold stress, while genes of alpha-linolenic acid metabolism pathway in GN were down-regulated. Induction of glycolysis/ gluconeogenesis pathways (ko00010) in DX leaves is essential to avoid energy starvation caused by reduced photosynthesis under late cold stress. Flavonoid biosynthesis (ko00941) and stilbenoid, diarylheptanoid and gingerol biosynthesis (ko00945) were significantly enriched in the DX and GN leaves exposed the prolonged cold stress. Majority genes involved in these processes were up-regulated in both genotypes, except genes of flavonoid biosynthesis in 24 h-treated GN leaves. The transcriptomes of the DX and GN leaves further displayed an enrichment of the category protein processing in endoplasmic reticulum (ko04141) in response to late cold stress (Additional file 18: Table S10).

### ABA metabolism and signalling in response to cold stress

**Fig. 8:**
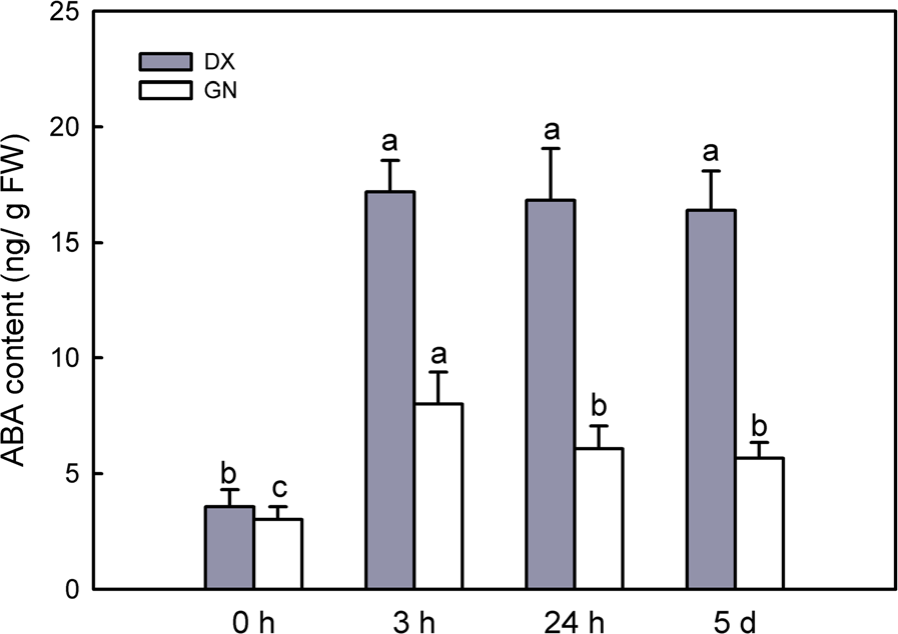
Changes in ABA content during the time course. Each value represents the mean of three replicates ± SE shown by the *vertical error bar*. The letters above the *bars* indicate a significant difference at the 0.05 level according to Duncan’s multiple range test

ABA is an important stress hormone that mediates abiotic stress responses in plants [21]. The genes involved in the ABA response increased over time in both genotypes. This is supported by the over-represented GO term ‘response to abscisic acid stimulus’ (GO:0009737) and ‘abscisic acid mediated signaling pathway’ (GO:0009738) seen after 5 d of cold treatment, especially in DX genotype (Additional file 24: Table S18). Six ABA synthesis-related genes, encoding zeaxanthin epoxidase and a probable aldehyde oxidase, were induced in both genotypes. ABA catabolism contained genes abscisic acid 8'-hydroxylase 3 (*CYP707A7*) and abscisate beta-glucosyltransferase (*AOG*) were exclusively induced in GN, while abscisic acid 8'-hydroxylase 1 (*CYP707A5*) declined only in DX. We further meaured the ABA content in both plants. ABA levels immediately increased in both genotypes in response to cold, with DX showing this effect to a greater extent (Fig. 8).

### Dehydrins function as candidate genes regulating cold tolerance

Numerous COR genes were induced during the cold stress in both genotypes (Additional file 25: Table S19). Most COR genes were expressed at common levels of expression, indicating these COR genes were conserved in regulating down-stream defense responses in both genotypes. Four genes encoding dehydrin (2, 3, and *RAB15*) and cold shock protein (*CSP1*) over-accumulated only in DX. While two late embryogenesis abundant protein-related genes (*LEA-like*, *LEA1*) were induced exclusively in GN. *DHN4* was induced in DX but repressed in GN at the later stages of cold stress. Genes encoding *LEA14-A*, cold-regulated 413 plasma membrane protein (*COR413PM*), dehydrin (*COR410*), and cold-responsive protein (*COR14a*) had higher transcriptional abundance in DX compared with GN, indicating those CORs may contribute to the enhanced cold tolerance in DX. In contrast, *DHN5* and *LEA3* were much stronger over-represented in GN than in DX (Additional file 25: Table S19).

### Co-expression network and regulatory interactions

In order to further clarify the regulatory network induced by cold stress in both genotypes, a co-expression network analysis was performed using Pearson correlation thresholds [22]. In total, 98 and 140 genes formed a cold-responsive co-expression network in DX and GN, respectively (Additional file 26: Figure S7; Additional file 27: Table S20). These networks were directly connected with each other via 1632 edges in DX and 1288 edges in GN, suggesting that the coexpressed genes are most likely coregulated. Twenty-six of these coexpressed genes (22 in DX, 4 in GN) were strongly interconnected, with each gene having more than 50 edges (Additional file 26: Figure S7). These genes were therefore defined as hub genes. These hub genes were directly connected to each other via 428 edges in DX and 12 edges in GN, forming a highly interconnected subnetwork. Twenty-two hub genes (84.6 %) were annotated as COR (*COR413PM1*, *COR410*, *DHN5*, etc.), reflecting their central roles in the acquisition of cold tolerance.

### Validation of gene expression profiles by qRT-PCR

To confirm the accuracy and reproducibility of this Illumina RNA-seq result, 26 DEGs including 10 hub genes were chosen for qRT-PCR. These genes were from various functional categories include signaling, transcript factor, metabolism, and cold-responsive protein. The primer sequences, FPKM and qRT-PCR result are listed in Additional file 28: Table S21 and Additional file 29: Figure S8. All 26 genes in the two genotypes showed the same expression patterns in the qRT-PCR assays as in the RNA-Seq data (Additional file 29: Figure S8). This independent evaluation revealed the reliability of the RNA-seq data.

## Discussion

The study provides a profile of the physiological and global transcriptomic response induced by cold stress in two *E. nutans* genotypes which display contrasting cold tolerance profiles, the wild DX and variety GN. Our physiochemical data demonstrated that cold stress resulted in more severe oxidative damage and growth suppression in GN than in DX, suggesting that DX is more cold-tolerant compared with GN. Proline acts as an osmolyte and the enhanced accumulation in plant cell lead to increased stress tolerance because of its biological function in retaining water in cells and reduce the rate of water loss in response to cold stress [23]. Consistent with this notion, higher proline accumulation and lower water loss observed in DX may account for the higher tolerance of DX to cold stress.

A whole-transcriptome analysis was performed in both cold-stressed and unstressed plants to obtain new insights into the molecular mechanisms of the cold stress response in both genotypes. For each sample from the two genotypes, more than 65 M high-quality clean reads were obtained, which were *de novo* assembled into 200,520 transcripts with a N50 of 1809 bp were generated in DX and 181,331 transcripts with a N50 of 1777 bp in GN, respectively, which indicates a high quality assembly that is likely to include many full-length cDNAs. A greater length of N50 in DX compared with GN may be the result from the larger number of longer fragment transcripts in DX. In addition, 82.20 % and 83.02 % of the assembled transcripts had homologs in at least one of the public databases that we searched, and 77.17 % and 77.15 % of the transcripts had a homolog that was determined with a high probability score in four species (*Hordeum vulgare subsp. Vulgare*, *Aegilops tauschii*, *Triticum urartu*, and *Brachypodium distachyon*) in DX and GN, respectively. Taken together, these results suggested that our *E. nutans* EST dataset represents a valuable transcriptome resource for gene discovery and functional analysis. It has been demonstrated that plants can enhance their cold tolerance by the induction or repression of gene transcripts in response to cold stress [15, 19]. In this study, a total of 5436 and 4323 DEGs were identified in DX and GN genotypes, respectively. Most of the cold-regulated genes are late-response genes, in agreemet with the reports by Lee et al. [15]. Approximately 18 % of the DX or GN DEGs had no annotated homologs in the public database, therefore these may be specific to *E. nutans* or represent cold-responsive genes with homologs that have not been identified in previous studies with other plant species.

### Different signaling and transcription factors mediate early cold stress responses

Plants under stress can trigger multiple signal transduction pathways that activate gene transcription and the concomitant downstream pathways responsible for physiological adaptation. Ca^2+^ is an important second messenger in plants and a key component in the signalling network by which plant cells respond to cold signals [6, 24]. Consistent with the expected role of Ca^2+^ in early cold signal transduction, we identified six up-regulated Ca^2+^ signaling-related genes at 3 h of cold stress. CBL proteins are known to interact with a group of serine/threonine protein kinases referred to as CIPKs [25]. The CBL–CIPK pathway has been reported to mediate plant responses to various environmental stresses, such as cold, drought and salinity [26]. The identification of *CIPK7* gene was exclusively induced in GN suggests that CBL-CIPK pathway may contribute to regulate cold response in the GN genotype. Additionally, plants can induce protein phosphorylation and dephosphorylation signals to withstand early cold stress. Our results showed that numerous protein kinases and phosphatases was the largest group among the signaling category in both genotypes, which is consistent with the resports by Lee et al. [15] in the Arabidopsis cold-responsive transcriptome.

Another prominent difference between the early cold responses of the *E. nutans* genotypes studied, was the large number of uniquely induced TFs. Most of these TFs belonged to the same families, such as AP2/EREBP, WRKYs, NACs, and MYBs. TFs play a key role in the regulation of upstream cold signals, which are capable of activating a cascade of downstream gene transcripts [16]. In the present study, the largest cold-inducible TF group belonged to the AP2/EREBP family and was composed of 35 members, which has been shown by some to be one of the best characterized CBFs in the cold regulatory pathway. The induced CBFs function to enhance cold tolerance in Arabidopsis and other plants [15, 27]. Our results therefore suggest that the *CBF* pathway is conserved in *E. nutans* responses to the cold stress. Other TFs including NAC, bHLH, bZIP, C2H2, WRKY, MYB, and HSF families were also exclusively induced in both genotypes. Previous studies have shown that members of these families function in cold tolerance [3, 12]. Moreover, three DX-specific TFs belonging to MADS, Sigma70-like, and Alfin-like subfamlies were differentially expressed, which may indicate that these TFs play important roles in coordinating genes involved in protective mechanisms in DX. Together, we speculate that the different TF transcripts in DX and GN may partially explain the different transcriptome changes induced by cold stress in the two genotypes.

### Cold stress signalling induces cellular protection processes

The complex interplay of different signaling pathways and multiple TFs co-ordinates the cellular response to cold stress that includes adjustments of the membrane system, cellular redox state, and primary and secondary metabolism [4, 16]. The effect of cold stress on cellular membrane properties is alleviated by membrane lipid restablishment. A group of lipid metabolism-related genes were already characterized in cold-treated DX and GN plants, including those encoding wax biosynthesis, cutin and suberin biosynthesis, sterol biosynthesis, fatty acid metabolism, and lipid transport and metabolism, were identified. Wax biosynthesis and deposition have been reported to be associated with the regulation of abiotic stresses tolerance [9, 28]. Oxylipin and wax might play important roles in the regulation of cold tolerance in both genotypes, indicated by high transcripts of their biosynthesis-related genes. A gene encoding fatty acid desaturase was strongly induced in DX and GN at 5 d under cold stress, which contributes to the protection of both plants from cold injury by regulating the fluidity of the cellular membrane [9]. These findings strongly suggest that lipid metabolism in E. nutans might be essential for the adaptation to cold stress.

ROS are frequently accumulated when plants suffered from environmental stress. ROS can function as a signal to trigger stress-related responses and they can also cause oxidative damage to plant cells [29]. Cold-treated DX and GN plants showed different ROS and MDA accumulation which is indicator of oxidative damage. To avoid the oxidative burst, plants possess a complex antioxidative defense system composed of nonenzymatic and enzymatic components. Genes encoding GST, POD, AsA-GSH cycle, Prx/Trx pathway, and PPO were detected in both plants; these genes have been shown to be involved in scavenging ROS under abiotic stress [30, 31]. Higher SOD, CAT, APX and GR activities were measured in cold-treated DX indicating that higher efficiency in the balance of ROS production. Furthermore, the transriptome results also showed the up-regulation of nonenzymic antioxidants in DX, including tocopherol, GSH, and polyamine, which have been reported to function in elevating stress tolerance [9, 32]. These results will help in broader understanding of the enhanced cold tolerance in DX partially resulted from ROS pathways modulation.

Sugar accumulation is a fundamental component of enhanced low temperature tolerance and acts in concert with the COR gene products [33]. Fructan, sucrose, fructose, and raffinose probably act as osmoprotectants to stabilize cellular membranes and a scavenger of ROS to protect plants against oxidative damage exposed to cold [13, 34, 35]. This was observed mainly in DX, where the greater accumulation of these sugars and higher induction of biosynthesis-related genes were over-represented at later phases of cold stress. Similar results have been reported in cold-treated perennial ryegrass [13]. The accumulation of trehalose is a protective strategy plants employ by using oxidative detoxification against environmental challenges [36]. Specific induction of *TPS6* in DX combined with higher induction of *TPP7* (a trehalose synthesis-related gene) in DX than in GN over the entire stress may reflect higher trehalose accumulation in the acquisition of cold tolerance.

In addition to activate primary mechanisms of ROS detoxification, the two plants also induce secondary mechanisms leading to the biosynthesis of other types of secondary metabolites. Flavonoids act as ROS scavengers that protect against oxidative damage under abiotic stress [20]. In this study, 56 flavonoid biosynthesis-related genes were detected and most of them responded late in the cold in both genotypes, with higher expression in DX than in GN. This result suggests that the activation of flavonoid metabolism at the late stage may be a adaptation mechanism of the plant to cold stress in *E. nutans*. This was consistent with the findings by Li et al*.* [37] who pointed out that the accumulated flavonoids protected petunias against cold damage. Similarly, Corso et al. [38] reported that flavonoid biosynthetic genes were induced in a drought-tolerant grapevine genotype. Additionally, other secondary metabolites biosynthesis-related genes such as those encoding DX-specific tropane alkaloid, caffeine, menthol, paclitaxel and GN-specific benzylisoquinoline alkaloid biosynthesis may play an important role in the prevention of oxidative injury against cold stress.

### Photosynthesis was inhibited under cold stress

Photosynthesis is a fundamental metabolic process for plant growth development, and this process is very sensitive to low temperatures [39]. In this study, we find that photosynthesis was inhibited by cold stress, and more related genes were down-regulated in GN than DX. Cold stress inhibited the transcription of chlorophyll biosynthesis-related genes and decreased chlorophyll content in both genotypes, but the decrease was greater in GN. Down-regulation of photosynthesis is affected by reduced activites of key photosynthesis-related enzymes under cold stress [40]. Three photosynthetic enzymes related genes encoding ribulose-1,5-bisphosphate carboxylase/oxygenase large subunit (*RbcL*) and ribulose bisphosphate carboxylase/oxygenase activase B (*RCAB*) showed exclusively differential expression in both plants. PSII was inhibited more severely in GN, as characterized by suppression of the genes encoding chlorophyll a/b binding proteins and the photosystem II reaction center *PSB28*. This agrees with the chlorophyll fluorescence data determined with PAM showing that DX maintained higher Fv/Fm, ETR, and lower NPQ relative to GN. The inhibition of photosynthesis-related genes under cold stress has also been observed in barley [41].

### ABA plays an important role in regulating cold tolerance

ABA functions as a stress hormone that regulates nearly 10 % of the protein-coding genes under various abiotic stress conditions [21]. A rapid increase in ABA content in both plants was observed at 3 h, but subsequently the levels remained constant with only slight changes in both plants. Interestingly, ABA synthesis-related genes were induced together with down-regulation of ABA catabolism in DX during the late of cold stress, which is consistent with the report by Yoshioka et al*.* [42]. Notably, numerous genes in the following categories ‘ABA-mediated signaling’ and ‘response to ABA stimulus’ were detected, which further confirmed that ABA-dependent signaling plays an important role in mediating adaptive responses in both plants.

### Dehydrins function as candidate genes regulating cold tolerance

The physiochemical responses for plant adaptation to cold stress results mainly from changes in the expression of COR genes. The LEA protein functions as an antioxidant and a membrane and protein stabilizer during cold stress [43]. The dehydrins, belonging to the class of LEA proteins have expression patterns highly correlated with cold-stress tolerance [4]. The levels of some of these proteins are controlled by CBF transcription factors [15]. In this study, several dehydrin-related genes over-accumulated in DX exclusively and contributed to the enhanced cold tolerance. Additionally, *LEA14-A*, *COR413PM*, *COR410*, and *COR14a* showed higher transcriptional abundance in DX than in GN, indicating less celluar membrane oxidative damage thereby enhancing cold tolerance in DX. Similar studies in wheat have shown that the expression of *Wcor410* and *Wcor14* was higher for cold-tolerant than cold-sensitive genotypes [44]. Combined with coexpression results, we speculate that the higher expression of COR genes enables DX to more effectively activate downstream defense reponses and further improve tolerance to cold stress.

## Conclusions

**Fig. 9:**
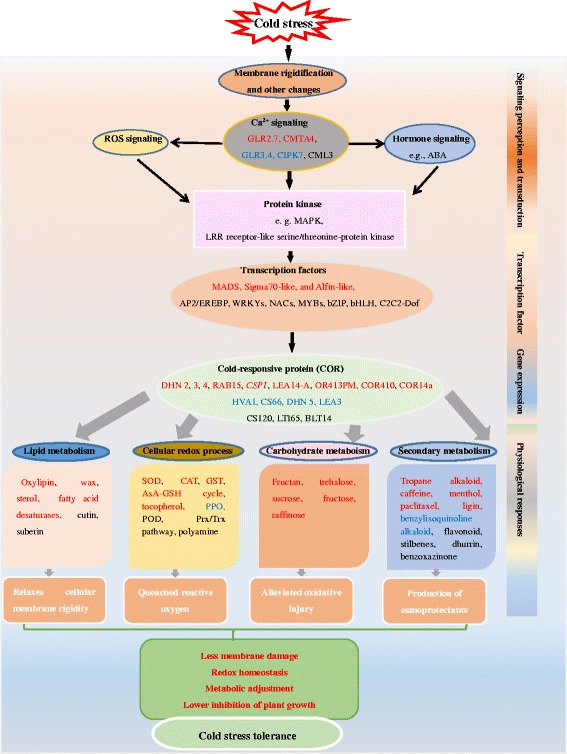
Hypothetical model summarizing the events occurring in leaves of DX and GN upon cold stress. *Red* text indicates DX specific and more highly expressed molecular responses. *Blue* and *black* text indicates GN specific responses and those common to both genotypes respectively

This study provides a comprehensive description of the transcriptomic responses to cold stress in the leaves of two *E. nutans* genotypes with different tolerances to cold stress. Based on physiochemical and transcriptomic data, a responsive model of cold stress in the two *E. nutans* could be summarized in Fig. 9. The cold signal is first perceived through cold-induced membrane rigidification and transduced further via Ca^2+^ signaling and protein kinases resulting in the activation of downstream TFs. The action of the TFs, trigger a cascade of downstream COR gene transcripts, whose activations further modulates cellular metabolic homeostasis and improved tolerance to cold stress. The stronger transcriptional differentiation during cold stress in DX explains its better cold tolerance compared to GN. The identified fructan biosynthesis, alpha-linolenic acid metabolism, and DX-specific dehydrin-related genes may provide genetic resources for the improvement of cold-tolerant characters in DX. The proposed model, which is based on transcription expression differences between two *E. nutans* genotypes, may facilitate future studies of the molecular mechanisms underlying cold stress responses in plants.

## Methods

### Plant materials and treatments

Seeds of *E. nutans* (Damxung, DX) were collected from wild plants growing in Damxung County, Tibet China (30°28.535′N, 91°06.246′E, altitude 4678 m). Seeds of *E. nutans* cultivar (Gannan, GN) were obtained from Lanzhou Xinglong Grass Industry Technology Service CO. Ltd and they were planted in Gannan (33°51.043′N, 101°40.139′E, altitude 2500 m), Gansu, China. The seeds were cleaned and stored at 4 °C in paper bags until the start of the experiments.

DX and GN seeds were surface-sterilized in 0.1 % (w/v) sodium hypochlorite, and germinated on moistened filter paper for 7 d at 25 °C. Morphologically uniform seedlings were transferred to pots using a 1 : 1 (v/v) mixture of vermiculite and sand as solid support. Five seedlings were planted in each pot and grown for 28 d in a growth chamber at a 25/25 °C day/night temperature, 14 h/10 h (day/night) photoperiod with a photon flux density of 350 μmol m^−2^ s^−1^ and a constant humidity of 70 %. Cold stress treatment was performed on these samples by transferring 28-d-old pot-growing plants (three-leaf stage) to a growth chamber set to 4 °C under the photoperiodic conditions described above [45]. The 28-d-old seedlings were exposed to cold stress at 09: 00 h, and leaf were harvested at 0 h (control), 3, 24 h, and 5 d after cold treatments, and then quickly frozen in liquid nitrogen and stored at –80 °C. Three biological replicates were performed at each time point. Each biological replicate consisted of a pool of leaves from five plants, grown in the same pot.

### Assay of dry weight and leaf water content

Three plants were randomly chosen from each group. The shoots of the seedlings were cut at the growth medium line. The shoots were dried at 80 °C for 72 h and their dry weights were determined. Relative water content (RWC) was measured according to Barrs and Weatherley [46].

### Determination of electrolyte leakage, lipid peroxidation and ROS Accumulation

Electrolyte leakage was determined by the modified method according to Song et al. [47]. The fresh leaves (0.5 g) were washed in deionized water and placed in Petri dishes with 5 ml deionized water at 25 °C for 2 h. After the incubation, the conductivity was measured (EC_1_). Then, the samples were boiled for 20 min and conductivity was read again (EC_2_). The electrolyte leakage was calculated as EC_1_/EC_2_ and expressed as percent.

Membrane lipid peroxidation was measured as the concentration of malondialdehyde (MDA) produced using 10 % (w/v) trichloroacetic acid (TCA), according to Dhindsa et al. [48]. The absorbance of the supernatant was measured at 450, 532, and 600 nm.

The concentration of H_2_O_2_ was measured by monitoring the absorbance of titanium-peroxide complex at 415 nm according to the method described by Shi et al. [49].

Superoxide radical production rate was determined by the plant O_2_^•−^ ELISA Kit based on antibody-antigen-enzyme-antibody complex following the manufacturer’s instruction [49].

### Measurement of antioxidant enzyme activities

The leaves (0.5 g) were homogenized with a mortar and pestle at 4 °C in 5 ml 50 mM phosphate buffer (pH 7.8) containing 1 mM EDTA and 2 % PVP. Homogenate was centrifuged at 12000 × g for 20 min at 4 °C and the supernatant was used for enzyme activity assays. Protein content in the supernatant was determined according to Bradford [50].

The assay for ascorbate peroxidase (APX) activity was measured according to Nakano and Asada [51]. The activity of catalase (CAT) was measured by following the consumption of H_2_O_2_ at 240 nm according to Cakmak and Marschner [52]. Glutathione reductase (GR) activity was measured by following the decrease in absorbance at 340 nm due to NADPH oxidation according to Shaedle and Bassham [53]. Activity of superoxide dismutase (SOD) was determined according to Beauchamp and Fridovich [54] by following the photo-reduction of nitroblue tetrazolium (NBT) at 560 nm.

### Assay of carbohydrate and proline contents

Contents of fructose, sucrose, and raffinose were determined using high performance liquid chromatography (Waters Corporation, America) as described by Liu et al. [55]. Total fructan contents were measured according to Rao et al. [56].

Leaves (0.1 g) were treated with 3 % (w/v) sulphosalicylic acid followed by boiling for 1 h. Proline contents were measured according to Bates et al. [57].

### Measurement of chlorophyll content and chlorophyll fluorescence parameters

Chlorophyll concentration was determined spectrophotometrically using 80 % acetone as a solvent [58]. Extract absorbance was measured at 645 and 663 nm with Optizen 5100 UV spectrophotometer (Shanghai, China). Maximal photochemical efficiency of PSII (Fv/Fm) apparent electron transport rate (ETR), non-photochemical quenching (NPQ) was determined using a PAM-2100 fluorometer (Walz, Effeltrich, Germany) as described by Oliveira and Peñuelas [59].

### Determination of ABA content

The ABA contents were measured using ultrahigh-performance liquid chromatography coupled to electrospray ionization tandem spectrometry (UHPLC/ESI-MS/MS) according to Müller and Munné-Bosch [60].

### Total RNA extraction, RNA-Seq library construction and sequencing

RNA samples were extracted from the leaves of three replicates of 0, 3, 24 h and 5 d cold-stress-treated DX and GN plants. Total RNA was extracted using a Trizol reagent according to the manufacturer’s protocol (Takara Bio Inc., Otsu, Japan). The integrity and purity of the RNA was determined by using the Agilent 2100 RNA 6000 Kit and electrophoresis on 1 % agarose gel.

Illumina sequencing was performed using Illumina HiSeq 4000 at the Beijing Genomics Institute (BGI)-Shenzhen, Shenzhen, China (http://www.genomics.cn/index) according to the manufacturer’s instructions (Illumina, San Diego, CA, USA) [61]. Briefly, mRNA was purified from 3 μg of total RNA using poly-T oligo-attached magnetic beads. Purified RNA was then fragmented at an elevated temperature using NEBNext First Strand Synthesis Reaction Buffer (NEB, Ipswich, MA, USA), targeting fragments range 200–500 bp. Fragmented RNA was purified using AMPure XP system (Beckman Coulter, Beverly, USA). Reverse transcription was performed using SuperScript II Reverse Transcription (Invitrogen, Shanghai). Double stranded cDNA fragments were purified and selected for targeted fragments (200–500 bp) using Ampure XP system. The cDNA was blunt-ended, poly-adenylated, and ligated with library adaptors using USER enzyme (NEB, USA). Digestion of dUTP was performed using AMPErase UNG (Applied Biosystems) to remove second strand cDNA. Digested cDNA was cleaned up with AMPure XP system. This was followed by amplification by 10 cycles PCR using NEBNext UltraTM RNA Library Prep Kit (NEB, Ipswich, MA, USA). The final library was cleaned up with AMPure XP system. Sequencing was done on the Illumina platform generating paired end reads of 100 bp each. Raw reads were filtered and trimmed based on quality and adapter inclusion using the SOAPnuke 1.5.0 software (http://soap.genomics.org.cn/) [62]. After filtering, the remaining reads are called ‘clean reads’ and stored in FASTQ. During the QC steps, Agilent 2100 Bioanaylzer and ABI StepOnePlus Real-Time PCR System were used in quantification and qualification of the sample library.

### *De novo* assembly and sequence clustering

The adapter sequences were removed, and raw reads containing ambiguous bases ‘N’ > 5 % or those what were low-quality (more than 20 % Q ≤ 10 bases) were removed. The Inchworm, Chrysalis, and Butterfly modules of Trinity software (version: v2.0.6) was used for *de novo* assembly of the clean reads [61]. The Inchworm program assembled the reads into the unique sequences of transcripts; Chrysalis program clusters the Inchworm Contigs into clusters and constructs complete de Bruijn graphs for each cluster. Finally, Butterfly program constructed the transcripts. Then use Tgicl v2.0.6 clustered transcripts to unigenes, which involves sequence splicing and redundancy removal to acquire non-redundant unigenes [63]. Putative protein translations were extracted using ESTScan (v3.0.3, default parameters) [64].

### Unigene function annotation

Unigene sequences were aligned by Blastx to protein databases such as Nr, Swiss-Prot, KEGG and COG and aligned by Blastn to the nucleotide database Nt (E-value, 0.00001). This was done to achieve the best functional annotations. With Nr annotation, we used the Blast2 GO program (version: v2.5.0) [65] to obtain the gene ontology (GO) annotation for the unigenes, and we used InterProScan5 (version: v5.11-51.0) [66] to get the InterPro annotation.

### Analysis of differentially expressed genes

For each sample, we mapped clean reads to Unigenes using Bowtie2 version v2.2.5 [67], and then calculated the gene expression levels using RSEM (version: v1.2.12) [68]. Fragments Per Kilobase of transcript per Million fragments mapped (FPKM) of each sequence for each transcriptome as measure of expression levels. Differentially expressed genes identified by NOIseq were required to have a 2-fold change and *P* ≥ 0.8 [69]. NOISeq is a non-parametric approach for the identification of differentially expressed genes from normalized count data. NOISeq empirically models the noise distribution of count changes by contrasting fold-change differences (M) and absolute expression differences (D) for all the features in samples within the same condition. This reference distribution is then used to assess whether the M-D values computed between two conditions for a given gene is likely to be part of the noise or represent a true differential expression. Hierarchical clusters were calculated using Gene-E (www.broadinstitute.org/cancer/software/GENE-E/) with the Pearson uncentred metric distance and an average linkage.

### GO term and KEGG pathway enrichment analysis

Based on the GO annotation result, gene ontology (GO) enrichment analysis of differentially expressed genes (DEGs) was conducted using the GOseq R package and correcting p-values with hypergeometric test [70]. Next, we performed KEGG pathway functional enrichment using phyper, a function within the R package. The FDR terms not larger than 0.001 were defined as significant enriched.

### Co-expression network analysis

Co-expression network analysis was performed using Pearson correlation thresholds (*r* ≥ 0.95, *P* ≤ 0.0001), as described by Mentzen et al. [22]. Hub genes were selected from the network (≥50 edges). Coexpression network were constructed using Cytoscape version v3.1.1 (http://www.cytoscape.org/).

### qRT-PCR validation

The purified RNA samples were reverse-transcribed using the PrimeScript RT Reagent Kit with gDNA Eraser (Takara, Dalian, China), following the manufacturer’s protocol. Twenty-six unigenes were selected randomly for the qRT-PCR assay. Gene specific qRT-PCR primers were designed using Primer3 software (http://primer3.ut.ee/). qRT-PCR was carried out using a LightCycler 480 RT-PCR system (Roche Applied Science, Germany). The primer pairs used in qRT-PCR reactions are listed in Table S19. *18S rRNA* was used as an internal control for expression analyses. Three replicates of each cDNA sample were performed for qRT-PCR analysis.

### Statistical analysis

Differences in physiological responses in the two genotypes were examined by one-way ANOVA and Duncan multiple comparison if ANOVA result was significant (*P* < 0.05). Statistical analysis was run by SPSS-17 statistical software (SPSS Inc., Chicago, IL, USA).

## Additional files

Additional file 1: Figure S1. Effect of cold stress on fresh weight (a), relative water content (b), electrolyte leakage levels (c), MDA content (d), and proline content (e) in DX and GN. Each value represents the mean of three replicates ± SE shown by the vertical error bar. Different letters above the bars indicate significant difference at the 0.05 level according to Duncan’s multiple range test. (PDF 319 kb)
Additional file 2: Table S1. Summary of transcripts assembly. (XLSX 10 kb)
Additional file 3: Figure S2. Length distribution of unigenes in DX and GN. (PDF 255 kb)
Additional file 4: Table S2. List of *Elymus nutans* transcriptome annotations. (XLSX 10 kb)
Additional file 5: Figure S3. The species distribution is shown as a percentage of the total homologous sequences with an E-value of at least 1.0E-5. (PDF 244 kb)
Additional file 6: Figure S4. Gene Ontology (GO) functional annotation of unigenes in DX and GN. (PDF 998 kb)
Additional file 7: Figure S5. KEGG Function classification results of unigenes in DX and GN. (PDF 435 kb)
Additional file 8: Table S3. Summary of annotation and expression of 5436 and 4323 differentially expressed genes (DEGs) in DX and GN under cold stress. (XLSX 1993 kb)
Additional file 9: Table S4. Annotation of 4074 DEGs between DX and GN in response to cold stress. (XLSX 1516 kb)
Additional file 10: Table S5. Specifically expressed genes in DX and GN at 3 h of cold stress compared with control (0 h). (XLSX 388 kb)
Additional file 11: Table S6. Specifically expressed genes in DX and GN at 24 h under cold stress compared control (0 h). (XLSX 61 kb)
Additional file 12: Table S7. Specifically expressed genes in DX and GN at 5 d under cold stress compared with control (0 h). (XLSX 139 kb)
Additional file 13: Table S8. Common expression DEGs in response to cold stress in DX and GN genotypes. Persistently up-regulated genes is highlighted in red and bold. (XLSX 85 kb)
Additional file 14: Table S9. The top 10 KEGG metabolic pathways enriched in DEGs. (XLSX 13 kb)
Additional file 15: Table S11. Differentially expressed transcription factors (TFs) in response to cold treatment of DX and GN genotypes. DX-specific TFs are color-coded with pale yellow shaded. GN-specific TFs are color-coded with light green shaded. Common expression TFs are color-coded with gray shaded. Significant (*P* > 0.8) decrease of transcript abundance is highlighted in blue and bold, significant transcript increase is highlighted in red and bold. (XLSX 490 kb)
Additional file 16: Table S13. Differently-expressed genes related to lipid metabolism in response to cold treatment of DX and GN genotypes. (XLSX 415 kb)
Additional file 17: Table S12. Differently-expressed genes related to signaling transduction during the early phases of cold stress. (XLSX 47 kb)
Additional file 18: Table S10. List of KEGG pathway enrichment analysis (Q value ≤ 0.05) of specifically expressed genes in DX and GN in response to 3, 24 h, and 5 d of cold stress. (XLSX 358 kb)
Additional file 19: Figure S6. Changes in the reactive oxygen accumulation and activities of antioxidative enzyme in DX and GN under cold stress. (PDF 308 kb)
Additional file 20: Table S14. Differently-expressed genes related to ROS producing and scavenging system in response to cold treatment of DX and GN genotypes. (XLSX 397 kb)
Additional file 21: Table S15. Differently-expressed genes related to carbohydrate metabolism in response to cold treatment of DX and GN genotypes. (XLSX 412 kb)
Additional file 22: Table S16. Differently-expressed genes related to secondary metabolism in response to cold treatment of DX and GN genotypes. (XLSX 415 kb)
Additional file 23: Table S17. Differently-expressed genes related to photosynthesis in response to cold treatment of DX and GN genotypes. (XLSX 352 kb)
Additional file 24: Table S18. Differently-expressed genes related to ABA metabolism and signaling in response to cold treatment of DX and GN genotypes. (XLSX 350 kb)
Additional file 25: Table S19. Differently-expressed genes related to cold-responsive protein in response to cold treatment of DX and GN genotypes. (XLSX 483 kb)
Additional file 26: Figure S7. The subnetwork of hub genes in DX (a) and GN (b). An edge indicates the coexpression between two genes. Red nodes represent up-regulated genes. (PDF 680 kb)
Additional file 27: Table S20. List of hub genes in in response to cold treatment of DX and GN genotypes. (XLSX 46 kb)
Additional file 28: Table S21. List of primer sequences of randomly selected unigenes used for qRT-PCR analysis. (XLSX 480 kb)
Additional file 29: Figure S8. Real time RT-PCR validation of differentially expressed transcripts from RNA-seq. Changes in the transcript levels of 16 specifically expressed genes in DX and GN genotype were shown in plot (a-h) and (i-p), respectively. Changes in the transcript levels of 10 hub genes in DX and GN genotype were shown in plot (q-v) and (w-z), respectively. Quantitative gene expression data are shown as the mean ± SE with three biological replicates. (PDF 1119 kb)

## Abbreviations

AP2/ERF: APETALA2/ETHYLENE RESPONSE FACTOR
bHLH: Helix–loop–helix
bZIP: Basic leucine zipper
CBF: C-repeat binding factor
DEGs: Differentially expressed genes
DX: Damxung
*E. nuatans*: *Elymus nutans*
ETR: Apparent electron transport rate
Fv/Fm: Maximum quantum efficiency of PSII
GN: Gannan
HCL: Hierarchical cluster analysis
NPQ: Non-photochemical quenching
ROS: Reactive oxygen species
TFs: Transcription factors

## References

[1] Wang WY, Wang QJ, Wang HC (2006). The effect of land management on plant community composition, species diversity, and productivity of alpine *Kobersia* steppe meadow. Ecol Res.

[2] Chen SY, Zhang XQ, Ma X, Huang LK. Assessment of genetic diversity and differentiation of *Elymus nutans* indigenous to Qinghai-Tibet Plateau using simple sequence repeats markers. Can J Plant Sci. 2013a; 93:1089–96

[3] Chinnusamy V, Zhu JK, Sunkar R (2010). Gene regulation during cold stress acclimation in plants. Methods Mol Biol.

[4] Thomashow MF (1999). Plant cold acclimation: freezing tolerance genes and regulatory mechanisms. Annu Rev Plant Biol.

[5] Xin Z, Browse J (2000). Cold comfort farm: the acclimation of plants to freezing temperatures. Plant Cell Environ.

[6] Reddy ASN, Ali GS, Celesnik H, Day IS (2011). Coping with stresses: roles of calcium- and calcium/calmodulin-regulated gene expression. Plant Cell.

[7] Mittler R (2002). Oxidative stress, antioxidants and stress tolerance. Trends Plant Sci.

[8] Hirayama T, Shinozaki K (2010). Research on plant abiotic stress responses in the post-genome era: past, present and future. Plant J.

[9] Maul P, McCollum GT, Popp M, Guy CL, Porat R (2008). Transcriptome profiling of grapefruit flavedo following exposure to low temperature and conditioning treatments uncovers principal molecular components involved in chilling tolerance and susceptibility. Plant Cell Environ.

[10] Winfield MO, Lu C, Wilson ID, Coghill JA, Edwards KJ (2010). Plant responses to cold: transcriptome analysis of wheat. Plant Biotechnol J.

[11] Chen SY, Huang X, Yan XQ, Liang Y, Wang YZ, Li XF, Peng X, Ma X, Zhang L, Cai Y, Ma T, Cheng L, Qi D, Zheng H, Yang X, Li X, Liu GS. Transcriptome analysis in sheepgrass (*Leymus chinensis*): a dominant perennial grass of the Eurasian Steppe. PLoS One. 2013b;8, e67974. 10.1371/journal.pone.0067974 PMC3701641 23861841

[12] Abeynayake SW, Byrne S, Nagy I, Jonavičienė K, Etzerodt TP, Boelt B, Asp T. Changes in *Lolium perenne* transcriptome during cold acclimation in two genotypes adapted to different climatic conditions. BMC Plant Biol. 2015a;15:250. 10.1186/s12870-015-0643-x PMC4609083 26474965

[13] Abeynayake SW, Etzerodt TP, Jonavičienė K, Byrne S, Asp T, Boelt B. Fructan metabolism and changes in fructan composition during cold acclimation in perennial ryegrass. Front Plant Sci. 2015b;6:329. 10.3389/fpls.2015.00329 PMC4428078 26029229

[14] Beike AK, Lang D, Zimmer AD, Wust F, Trautmann D, Wiedemann G, Beyer P, Decker EL, Reski R (2015). Insights from the cold transcriptome of *Physcomitrella patens*: global specialization pattern of conserved transcriptional regulators and identification of orphan genes involved in cold acclimation. New Phytol.

[15] Lee BH, Henderson DA, Zhu JK (2005). The Arabidopsis cold-responsive transcriptome and its regulation by ICE1. Plant Cell.

[16] Chinnusamy V, Zhu J, Zhu JK (2007). Cold stress regulation of gene expression in plants. Trends Plant Sci.

[17] Tommasini L, Svensson JT, Rodriguez EM, Wahid A, Malatrasi M, Kato K, Wanamaker S, Resnik J, Close TJ (2008). Dehydrin gene expression provides an indicator of low temperature and drought stress: transcriptome-based analysis of Barley (*Hordeum vulgare* L.). Funct Integr Genomics.

[18] Monteropalmero MB, Martinbarranco A, Escobar C, Hernandez LE (2014). Early transcriptional responses to mercury: a role for ethylene in mercury-induced stress. New Phytol.

[19] Janská A, Marík P, Zelenková S, Ovesná J (2010). Cold stress and acclimation – what is important for metabolic adjustment?. Plant Biol.

[20] Nakabayashi R, Yonekura-Sakakibara K, Urano K, Suzuki M, Yamada Y, Nishizawa T, Matsuda F, Kojima M, Sakakibara H, Shinozaki K, Michael AJ, Tohge T, Yamazaki M, Saito K (2014). Enhancement of oxidative and drought tolerance in Arabidopsis by overaccumulation of antioxidant flavonoids. Plant J.

[21] Sreenivasulu N, Harshavardhan VT, Govind G, Seiler C, Kohli A (2012). Contrapuntal role of ABA: does it mediate stress tolerance or plant growth retardation under long-term drought stress?. Gene.

[22] Mentzen WI, Peng JL, Ransom N, Nikolau BJ, Wurtele ES (2008). Articulation of three core metabolic processes in *Arabidopsis*: fatty acid biosynthesis, leucine catabolism and starch metabolism. BMC Plant Biol.

[23] Bartels D, Sunkar R (2005). Drought and salt tolerance in plants. Crit Rev Plant Sci.

[24] Galon Y, Finkler A, Fromm H (2010). Calcium-regulated transcription in plants. Mol Plant.

[25] Kim KN, Cheong YH, Gupta R, Luan S (2000). Interaction specificity of Arabidopsis calcineurin B-like calcium sensors and their target kinases. Plant Physiol.

[26] Luan S (2009). The CBL-CIPK network in plant calcium signaling. Trends Plant Sci.

[27] Nakashima K, Ito Y, Yamaguchi-Shinozaki K (2009). Transcriptional regulatory networks in response to abiotic stresses in Arabidopsis and grasses. Plant Physiol.

[28] Verelst W, Bertolinid E, De Bodt S, Vandepoele K, Demeulenaere M, Pèd ME, Inzéa D (2013). Molecular and physiological analysis of growth-limiting drought stress in brachypodium distachyon leaves. Mol Plant.

[29] Hernandez M, Fernandez-Garcia N, Diaz-Vivancos P, Olmos E (2010). A different role for hydrogen peroxide and the antioxidative system under short and long salt stress in *Brassica oleracea* roots. J Exp Bot.

[30] Garcia de la Garma J, Fernandez-Garcia N, Bardisi E, Pallol B, Salvador Asensio-Rubio J, Bru R, Olmos E (2015). New insights into plant salt acclimation: the roles of vesicle trafficking and reactive oxygen species signalling in mitochondria and the endomembrane system. New Phytol.

[31] Leng XP, Jia HF, Sun X, Shangguan LF, Mu Q, Wang BJ, Fang JG (2015). Comparative transcriptome analysis of grapevine in response to copper stress. Sci Rep.

[32] Huang XS, Zhang QH, Zhu DX, Fu XZ, Wang M, Zhang Q, Moriguchi T, Liu JH (2015). ICE1 of Poncirus trifoliata functions in cold tolerance by modulating polyamine levels through interacting with arginine decarboxylase. J Exp Bot.

[33] Gusta LV, Trischuk R, Weiser CJ (2005). Plant cold acclimation: the role of Abscisic acid. J Plant Growth Reg.

[34] Zeng Y, Yu J, Cang J, Liu LJ, Mu YC, Wang JH, Zhang D (2011). Detection of sugar accumulation and expression levels of correlation key enzymes in winter wheat (*Triticum aestivum*) at low temperatures. Biosci Biotech Bioch (BBB).

[35] Yue C, Cao HL, Wang L, Zhou YH, Huang YT, Hao XY, Wang YC, Wang B, Yang YJ, Wang XC (2015). Effects of cold acclimation on sugar metabolism and sugar-related gene expression in tea plant during the winter season. Plant Mol Biol.

[36] Li HW, Zang BS, Deng XW, Wang XP (2011). Overexpression of the trehalose-6-phosphate synthase gene *OsTPS1* enhances abiotic stress tolerance in rice. Planta.

[37] Li B, Ning LY, Zhang JW, Bao MZ, Zhang W (2015). Transcriptional profiling of Petunia seedlings reveals candidate regulators of the cold stress response. Front Plant Sci.

[38] Corso M, Vannozzi A, Maza E, Vitulo N, Meggio F, Pitacco A, Telatin A, D'Angelo M, Feltrin E, Negri AS, Prinsi B, Valle G, Ramina A, Bouzayen M, Bonghi C, Lucchin M (2015). Comprehensive transcript profiling of two grapevine rootstock genotypes contrasting in drought susceptibility links the phenylpropanoid pathway to enhanced tolerance. J Exp Bot.

[39] Zheng YL, Feng YL, Lei YB, Yang CY (2009). Different photosynthetic responses to night chilling among twelve populations of Jatropha curcas. Photosynthetica.

[40] Allen DJ, Ort DR (2001). Impacts of chilling temperature on photosynthesis in warm-climate plants. Trends Plant Sci.

[41] Svensson JT, Crosatti C, Campoli C, Bassi R, Stanca AM, Close T, Cattivelli L (2006). Transcriptome analysis of cold acclimation in barley Albina and Xantha mutants. Plant Physiol.

[42] Yoshioka T, Endo T, Satoh S (1998). Restoration of seed germination at supraoptimal temperatures by fluridone, an inhibitor of abscisic and biosynthesis. Plant Cell Physiol.

[43] Tunnacliffe A, Wise MJ (2007). The continuing conundrum of the LEA proteins. Sci Nat.

[44] Ganeshan S, Vitamvas P, Fowler B, Chibbar RN (2008). Quantitative expression analysis of selected COR genes reveals their differential expression in leaf and crown tissues of wheat (*Triticum aestivum* L.) during an extended low temperature acclimation regimen. J Exp Bot.

[45] Gao Q, Li X, Jia J, Zhao P, Liu P, Liu Z, Ge L, Chen S, Qi D, Deng B, Lee BH, Liu G, Cheng L (2016). Overexpression of a novel cold-responsive transcript factor *LcFIN1* from sheepgrass enhances tolerance to low-temperature stress in transgenic plants. Plant Biotechnol J.

[46] Barrs HD, Weatherley PE (1962). A re-examination of the relative turgidity techniques for estimating water deficits in leaves. Aust J Bio Sci.

[47] Song L, Ding W, Zhao M, Sun B, Zhang L (2006). Nitric oxide protects against oxidative stress under heat stress in the calluses from two ecotypes of reed. Plant Sci.

[48] Dhindsa RS, Plumb-dhindsa P, Thorpe TA (1981). Leaf senescence: correlated with increased levels of membrane permeability and lipid peroxidation and decreased levels of superoxide dismutase and catalase. J Exp Bot.

[49] Shi H, Ye T, Chen F, Cheng Z, Wang Y, Yang P, Zhang Y, Chan ZL (2013). Manipulation of arginase expression modulate abiotic stress tolerance in Arabidopsis: effect on arginine metabolism and ROS accumulation. J Exp Bot.

[50] Bradford MM (1976). A rapid and sensitive method for the quantitation of microgram quantities of protein utilizing the principle of protein-dye binding. Anal Biochem.

[51] Nakano Y, Asada K (1981). Hydrogen peroxide is scavenged by ascorbate-specific peroxidase in spinach chloroplast. Plant Cell Physiol.

[52] Cakmak I, Marschner H (1992). Magnesium deficiency and high light intensity enhance activities of superoxide dismutase, ascorbate peroxidase, and glutathione reductase in bean leaves. Plant Physiol.

[53] Shaedle M, Bassham JA (1977). Chloroplast glutathione reductase. Plant Physiol.

[54] Beauchamp C, Fridovich I (1971). Superoxide dismutase: improved assays and an assay applicable to acrylamide gels. Anal Biochem.

[55] Liu F, Jensen CR, Andersen MN (2004). Drought stress effect on carbohydrate concentration in soybean leaves and pods during early reproductive development: its implication in altering pod set. Field Crop Res.

[56] Rao RSP, Andersen JR, Dionisio G, Boelt B (2011). Fructan accumulation and transcription of candidate genes during cold acclimation in three varieties of Poa pratensis. J Plant Physiol.

[57] Bates LS, Waldren RP, Teare ID (1973). Rapid determination of free proline for water-stress studies. Plant Soil.

[58] Lichtenthaler HK (1987). Chlorophylls and carotenoids: pigments of photosynthetic biomembranes. Method Enzymol.

[59] Oliveira G, Peñuelas J (2004). Effects of winter cold stress on photosynthesis and photochemical efficiency of PSII of the Mediterranean *Cistus albidus* L. and *Quercus ilex* L. Plant Ecol.

[60] Müller M, Munné-Bosch S (2011). Rapid and sensitive hormonal profiling of complex plant samples by liquid chromatography coupled to electrospray ionization tandem mass spectrometry. Plant Methods.

[61] Grabherr MG, Haas BJ, Yassour M, Levin JZ, Thompson DA, Amit I, Adiconis X, Fan L, Raychowdhury R, Zeng Q, Chen Z, Mauceli E, Hacohen N, Gnirke A, Rhind N, di Palma F, Birren BW, Nusbaum C, Lindblad-Toh K, Friedman N, Friedman N (2011). Full-length transcriptome assembly from RNA-Seq data without a reference genome. Nat Biotechnol.

[62] Wang YM, Ding Y, Yu DW, Xue W, Liu JY (2015). High-throughput sequencing-based genome-wide identification of microRNAs expressed in developing cotton seeds. Sci China Life Sci.

[63] Pertea G, Huang XQ, Liang F, Antonescu V, Sultana R, Karamycheva S, Lee Y, White J, Cheung F, Parvizi B, Tsai J, Quackenbush J (2003). TIGR Gene Indices clustering tools (TGICL): a software system for fast clustering of large EST datasets. Bioinformatics.

[64] Iseli C, Jongeneel CV, Bucher P. ESTScan: a program for detecting, evaluating, and reconstructing potential coding regions in EST sequences. Intell Syst Mol Biol*.* 1999;138–48 10786296

[65] Conesa A, Götz S, García-Gómez JM, Terol J, Talón M, Robles M (2005). Blast2GO: a universal tool for annotation, visualization and analysis in functional genomics research. Bioinformatics.

[66] Quevillon E, Silventoinen V, Pillai S, Harte N, Mulder N, Apweiler R, Lopez R (2005). InterProScan: protein domains identifier. Nucleic Acids Res.

[67] Langmead B, Salzberg SL (2012). Fast gapped-read alignment with Bowtie 2. Nat Methods.

[68] Li B, Dewey CN (2011). RSEM: accurate transcript quantification from RNA-Seq data with or without a reference genome. BMC Bioinformatics.

[69] Tarazona S, Furió-Tarí P, Turrà D, Pietro AD, Nueda MJ, Ferrer A, Conesa A (2015). Data quality aware analysis of differential expression in RNA-seq with NOISeq R/Bioc package. Nucleic Acids Res.

[70] Young MD, Wakefield MJ, Smyth GK, Oshlack A (2010). Method gene ontology analysis for RNA-seq: accounting for selection bias. Genome Biol.

