# Supplementary figures and images for: De novo transcriptome sequencing and gene expression profiling of Elymus nutans under cold stress
Source: BMC Genomics. 2016 Nov 4;17:870. doi: 10.1186/s12864-016-3222-0 (PMC5097361; doi:10.1186/s12864-016-3222-0)

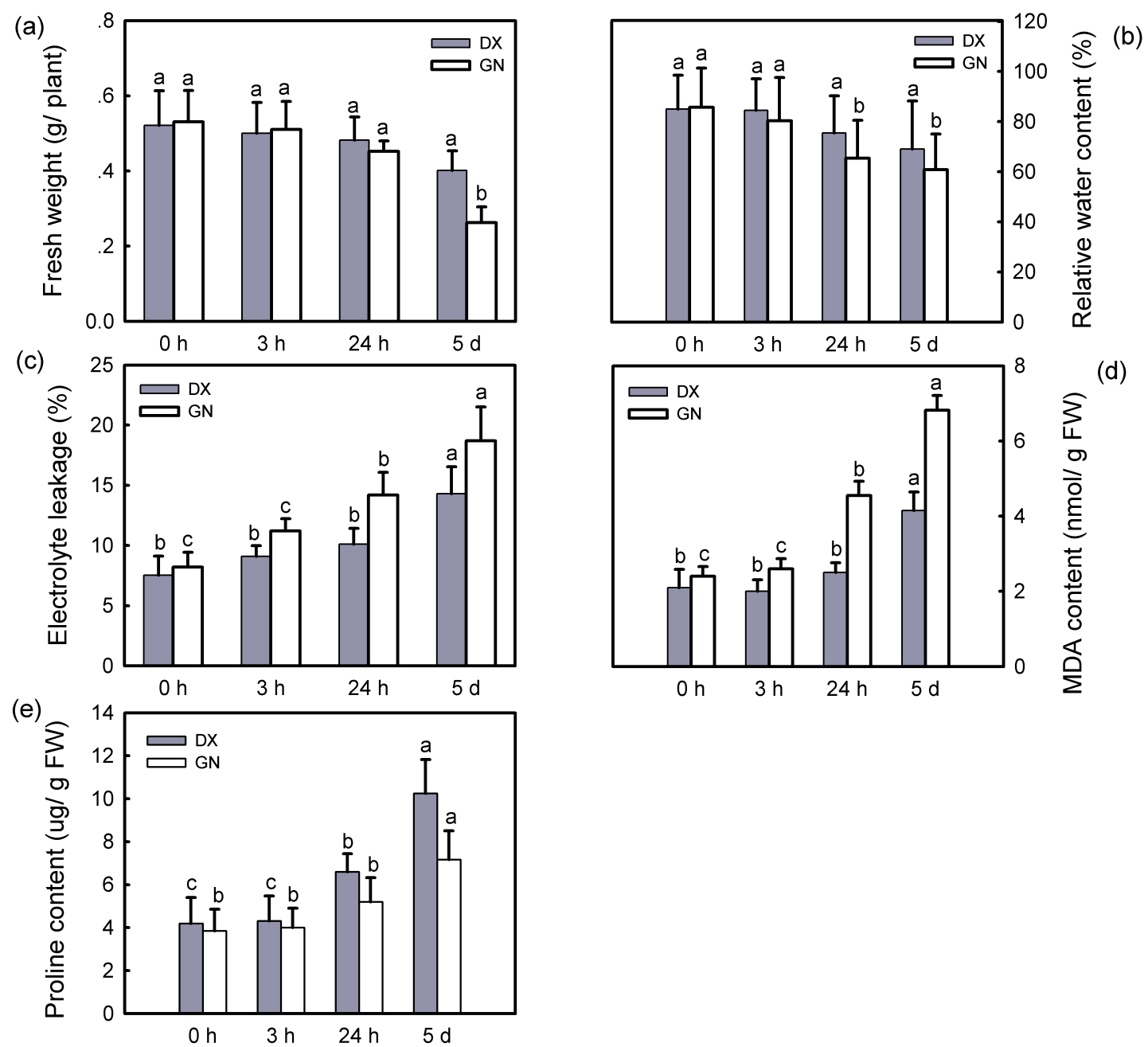

Figure S1

Supplement: Additional file 1: Figure S1. — Effect of cold stress on fresh weight (a), relative water content (b), electrolyte leakage levels (c), MDA content (d), and proline content (e) in DX and GN. Each value represents the mean of three replicates ± SE shown by the vertical error bar. Different letters above the bars indicate significant difference at the 0.05 level according to Duncan’s multiple range test. (PDF 319 kb) [file 12864_2016_3222_MOESM1_ESM.pdf]

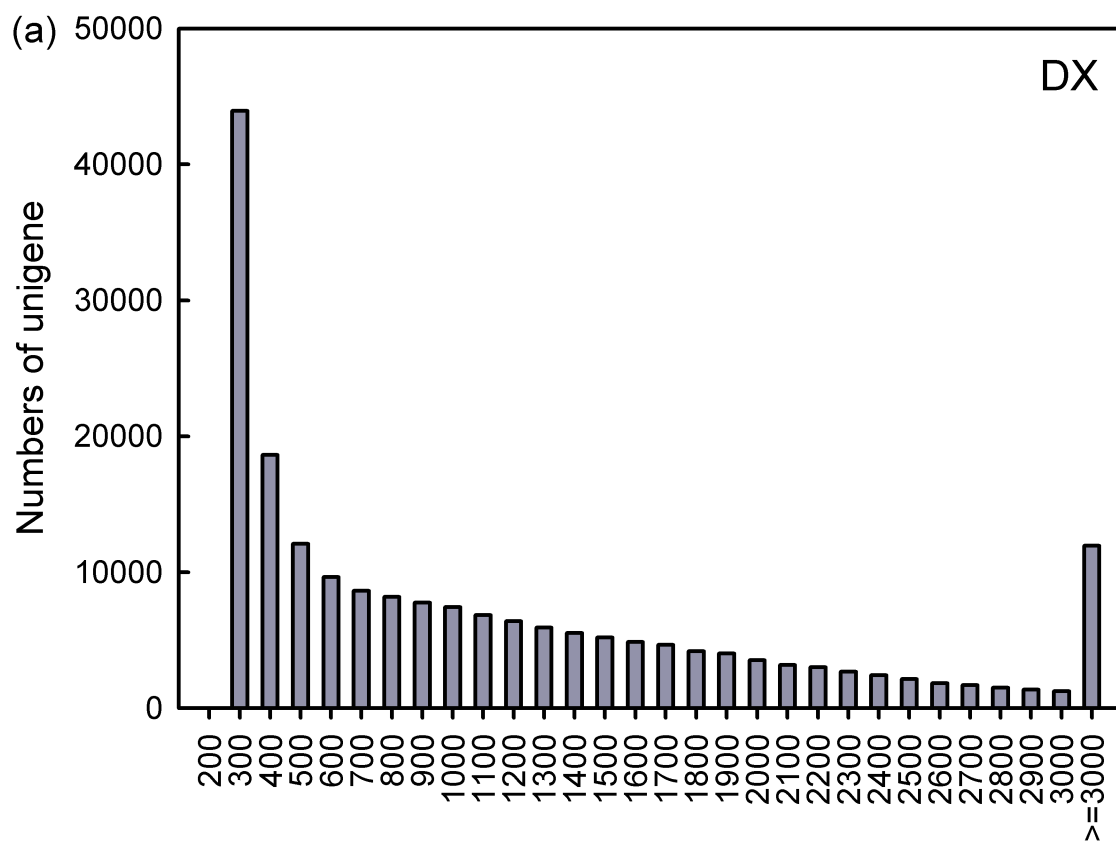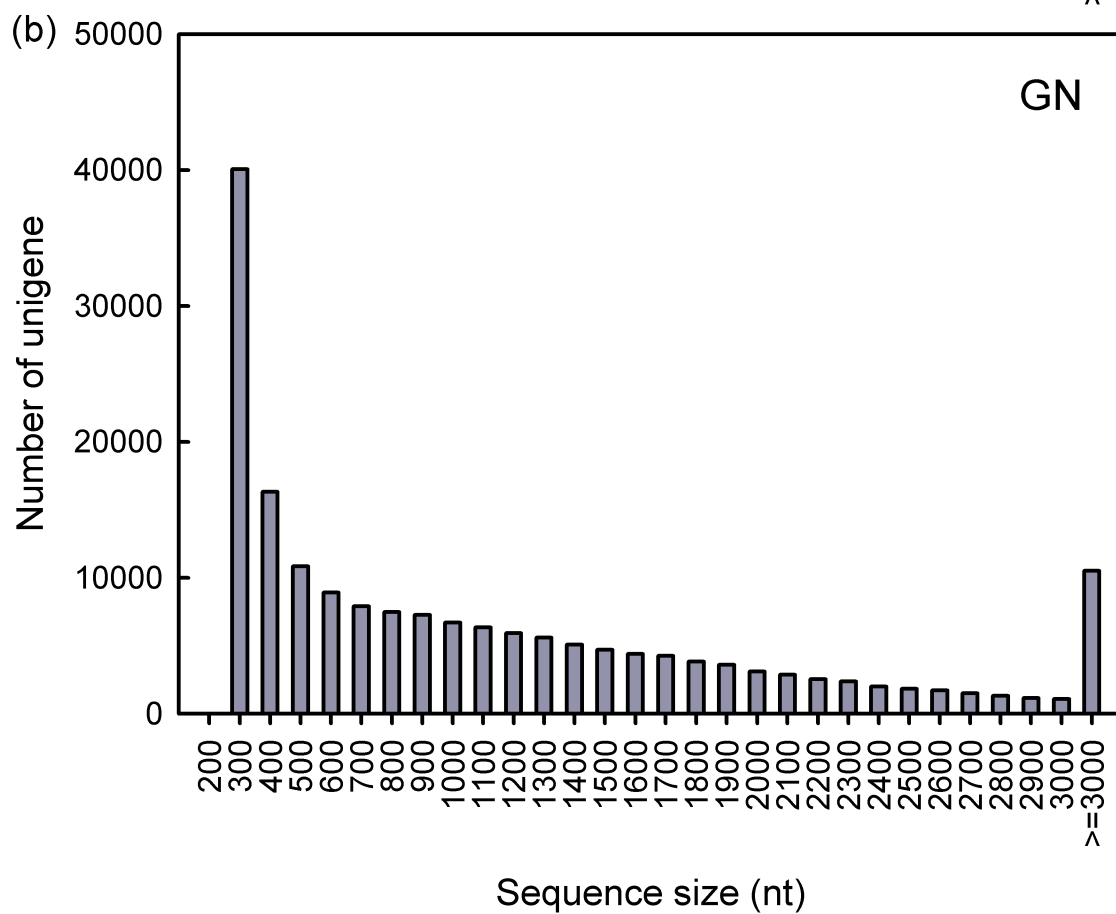

Figure S2

Supplement: Additional file 3: Figure S2. — Length distribution of unigenes in DX and GN. (PDF 255 kb) [file 12864_2016_3222_MOESM3_ESM.pdf]

(a) DX      Species Distribution

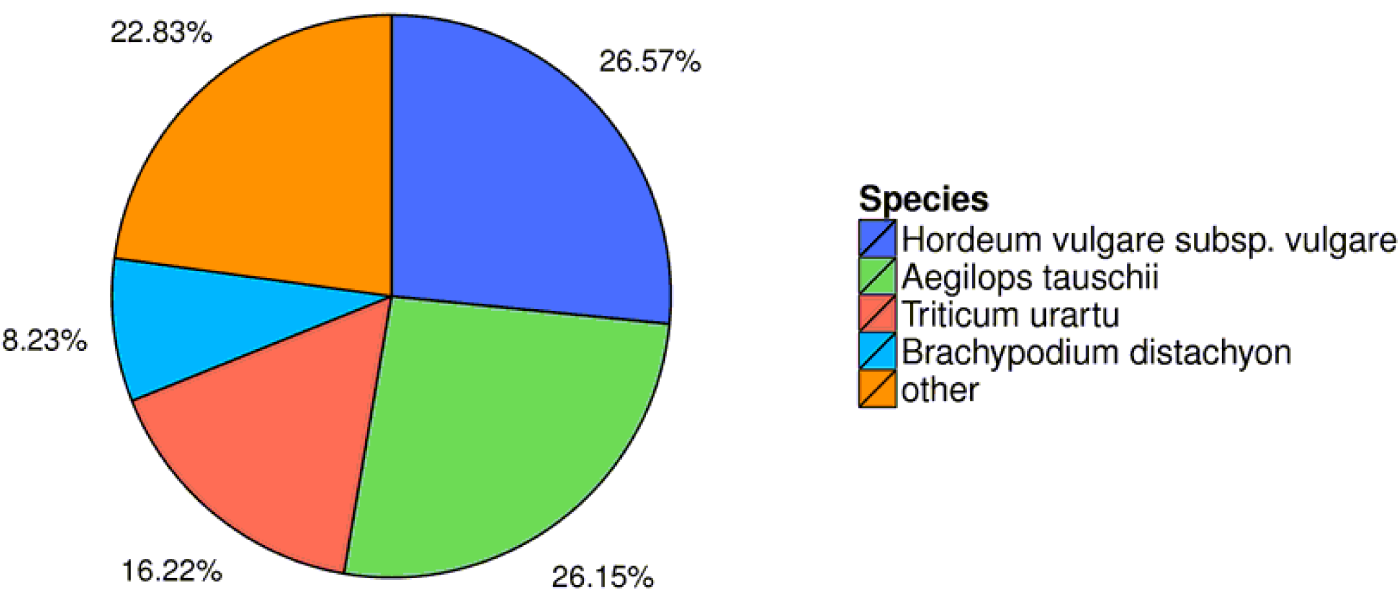

(b) GN      Species Distribution

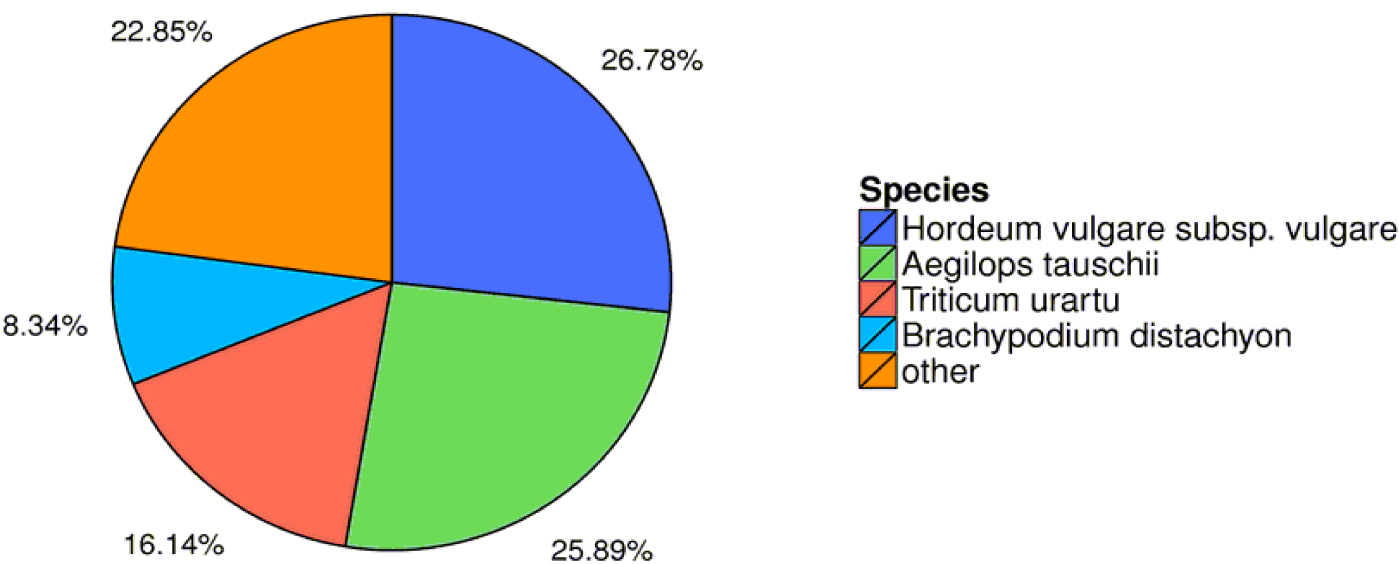

Figure S3

Supplement: Additional file 5: Figure S3. — The species distribution is shown as a percentage of the total homologous sequences with an E-value of at least 1.0E-5. (PDF 244 kb) [file 12864_2016_3222_MOESM5_ESM.pdf]

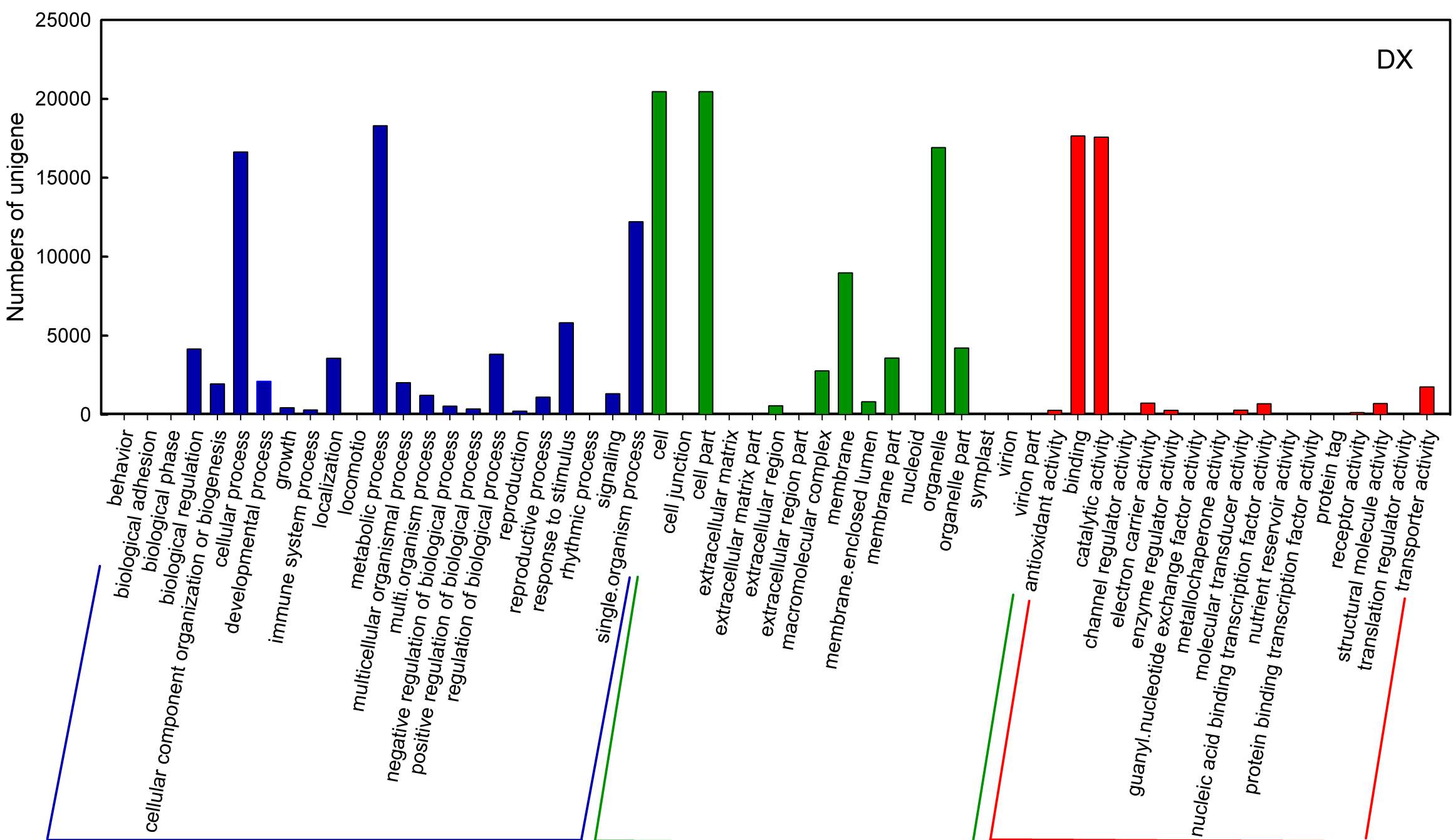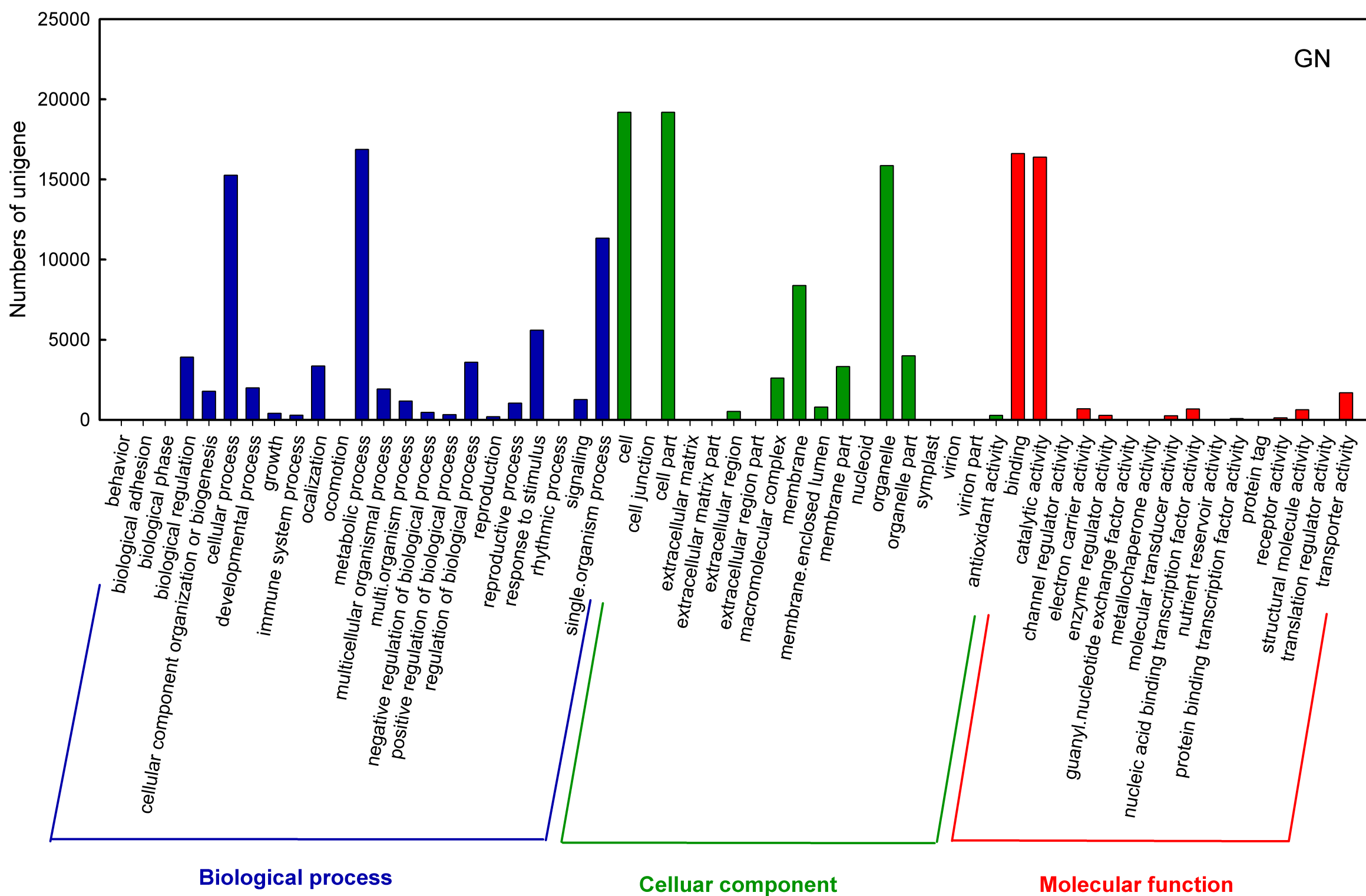

Figure S4

Supplement: Additional file 6: Figure S4. — Gene Ontology (GO) functional annotation of unigenes in DX and GN. (PDF 998 kb) [file 12864_2016_3222_MOESM6_ESM.pdf]

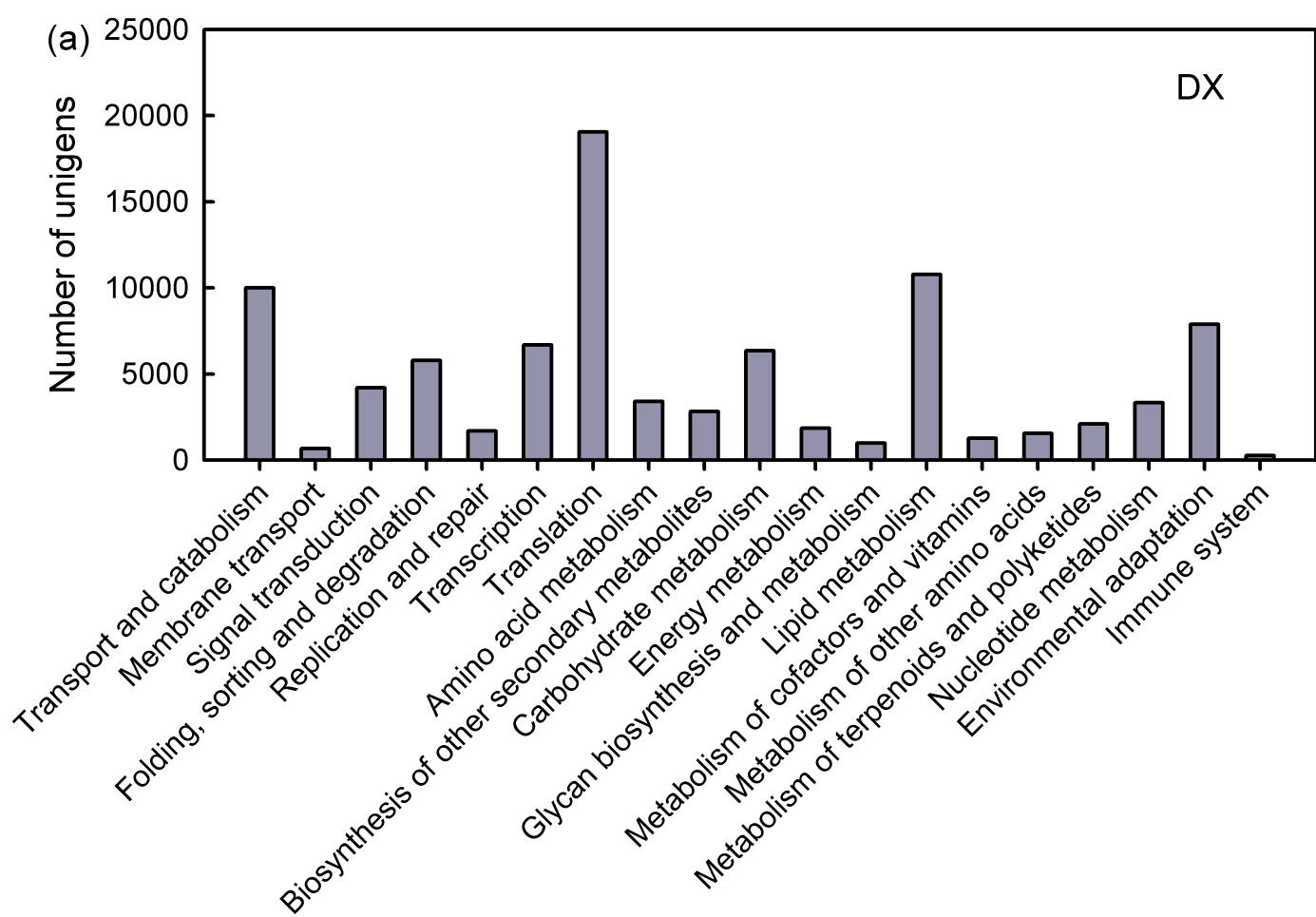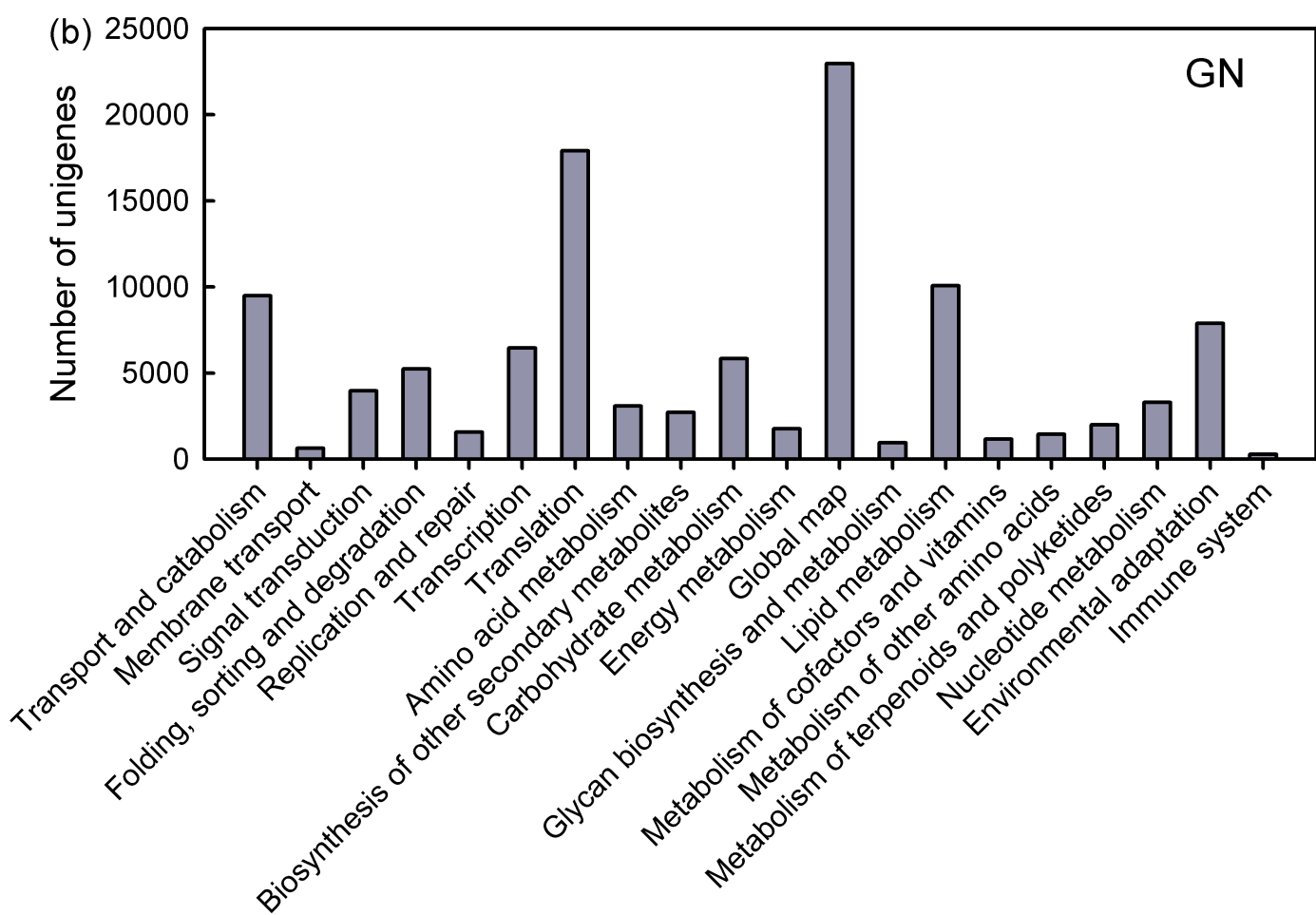

Figure S5

Supplement: Additional file 7: Figure S5. — KEGG Function classification results of unigenes in DX and GN. (PDF 435 kb) [file 12864_2016_3222_MOESM7_ESM.pdf]

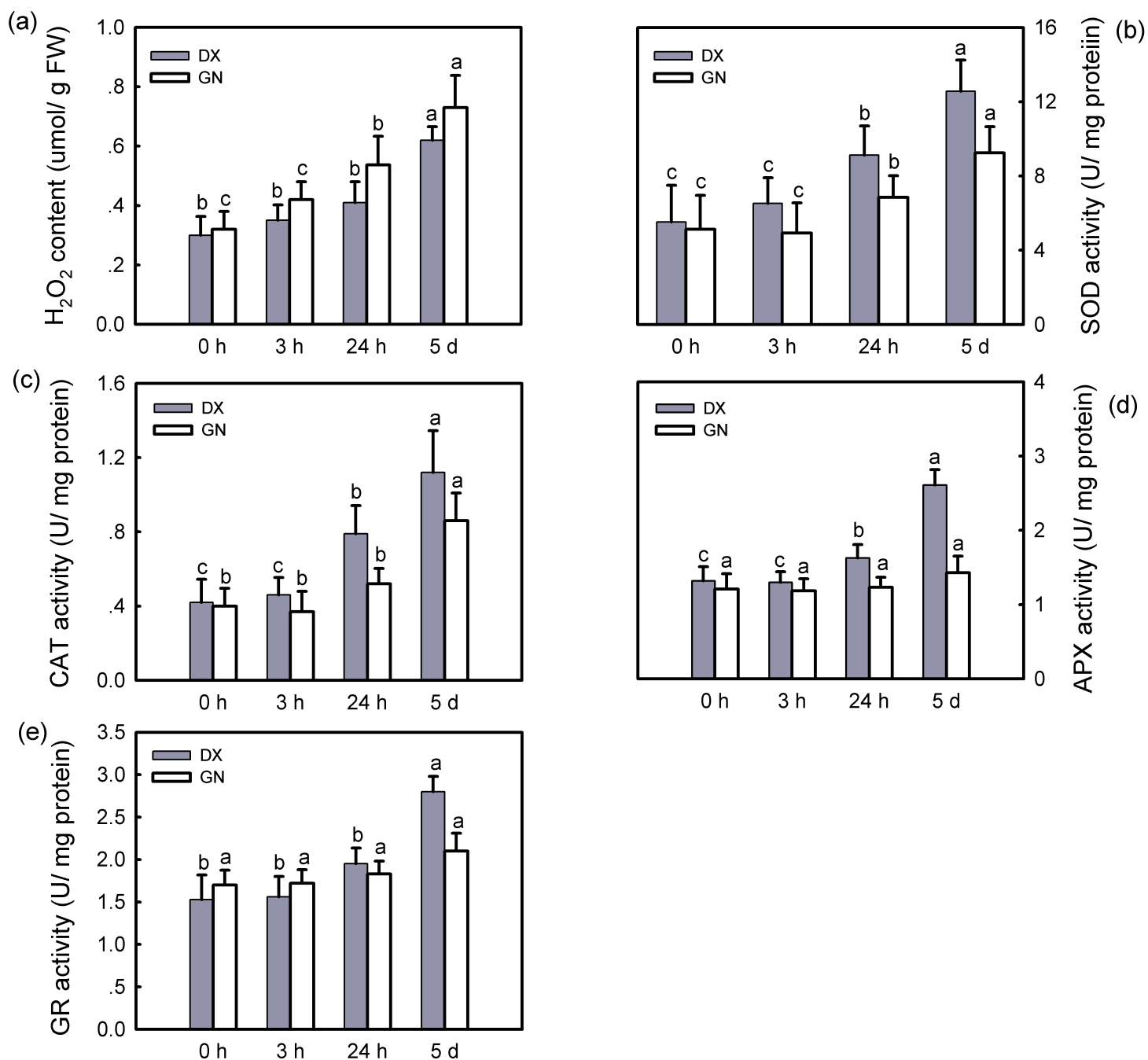

Figure S6

Supplement: Additional file 19: Figure S6. — Changes in the reactive oxygen accumulation and activities of antioxidative enzyme in DX and GN under cold stress. (PDF 308 kb) [file 12864_2016_3222_MOESM19_ESM.pdf]

(a) DX

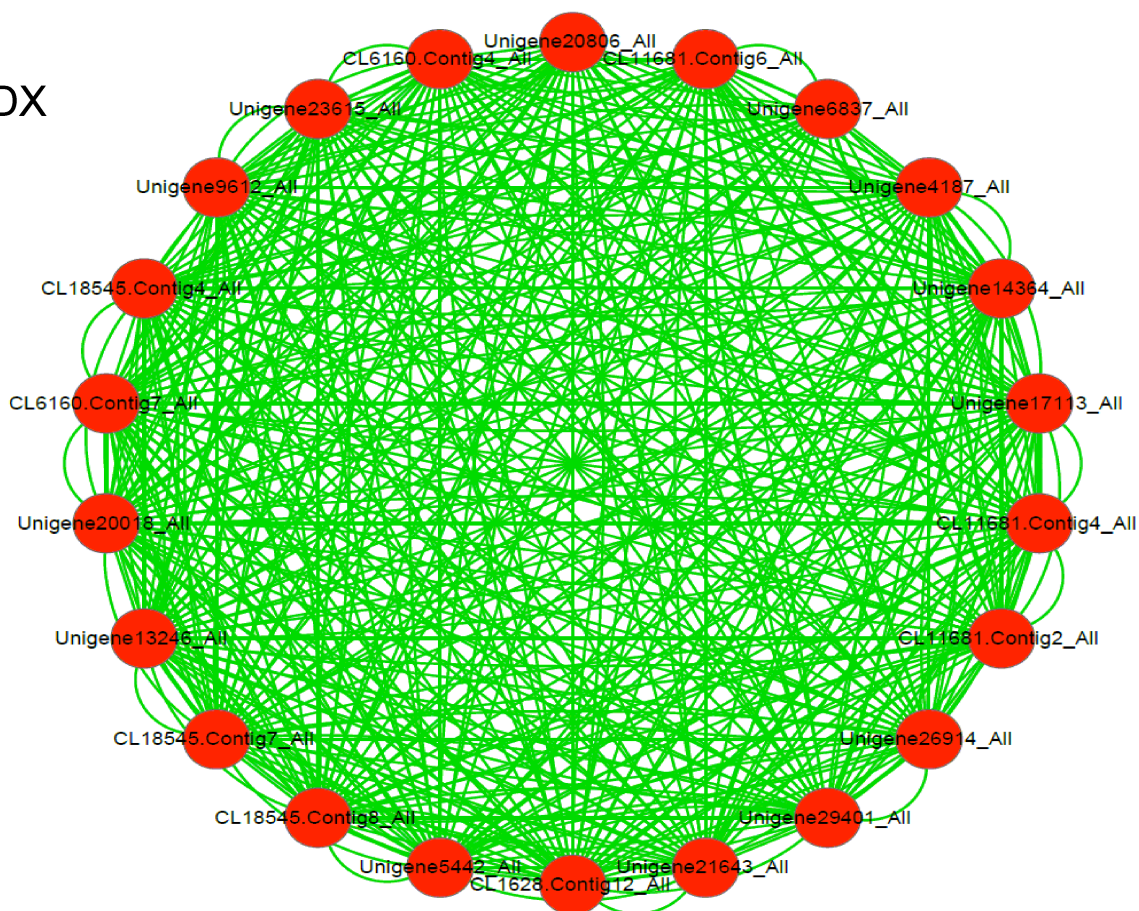

(b) GN

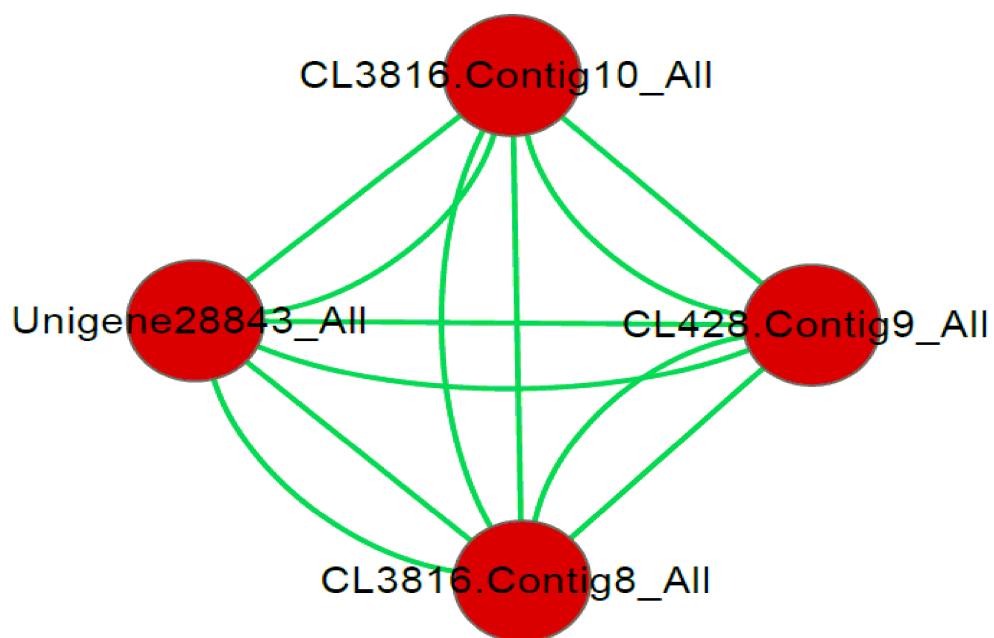

Figure S7

Supplement: Additional file 26: Figure S7. — The subnetwork of hub genes in DX (a) and GN (b). An edge indicates the coexpression between two genes. Red nodes represent up-regulated genes. (PDF 680 kb) [file 12864_2016_3222_MOESM26_ESM.pdf]

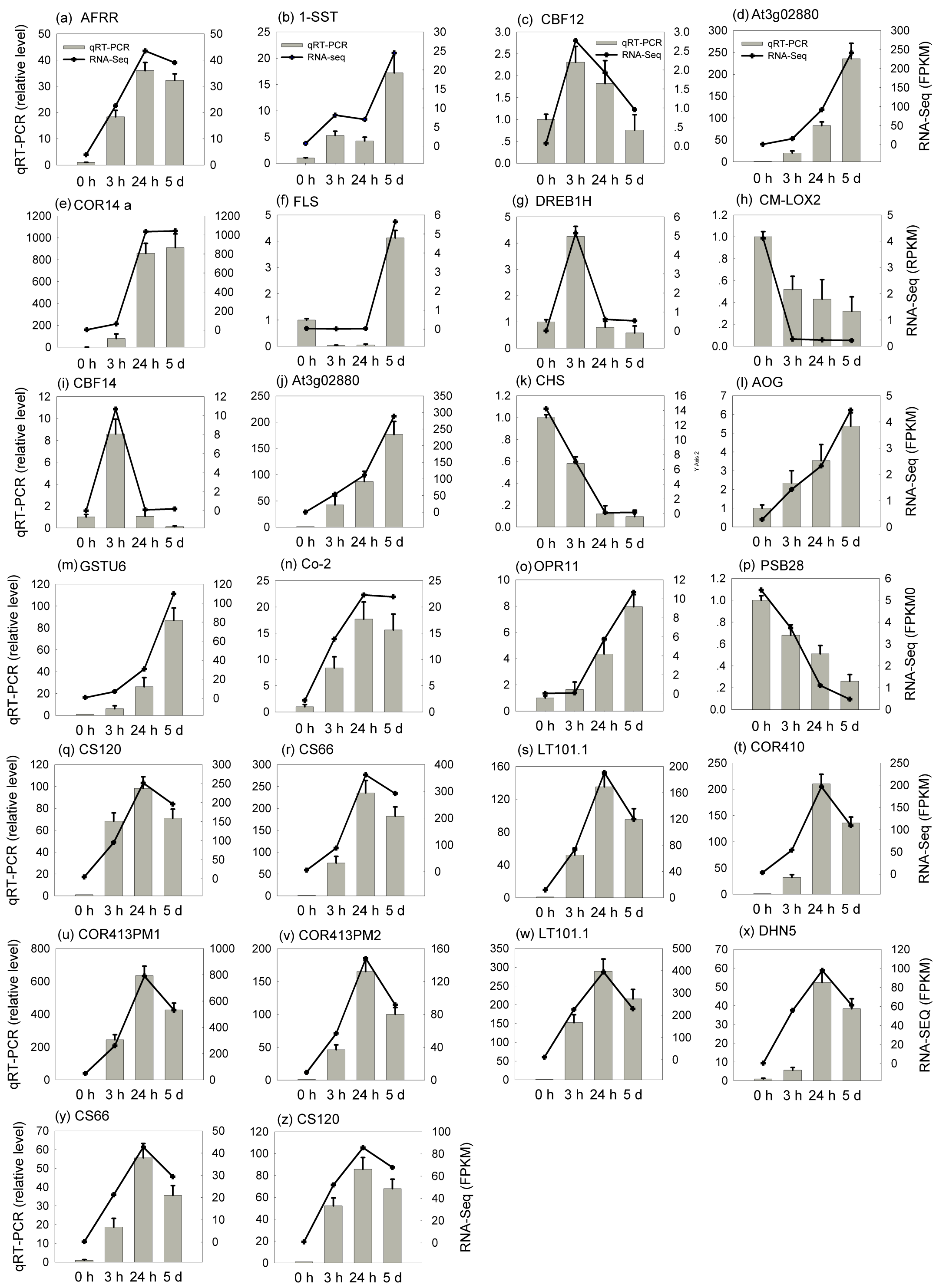

Figure S8

Supplement: Additional file 29: Figure S8. — Real time RT-PCR validation of differentially expressed transcripts from RNA-seq. Changes in the transcript levels of 16 specifically expressed genes in DX and GN genotype were shown in plot (a-h) and (i-p), respectively. Changes in the transcript levels of 10 hub genes in DX and GN genotype were shown in plot (q-v) and (w-z), respectively. Quantitative gene expression data are shown as the mean ± SE with three biological replicates. (PDF 1119 kb) [file 12864_2016_3222_MOESM29_ESM.pdf]
